# Sex is determined by XY chromosomes across the radiation of dioecious *Nepenthes* pitcher plants

**DOI:** 10.1002/evl3.142

**Published:** 2019-10-01

**Authors:** Mathias Scharmann, T. Ulmar Grafe, Faizah Metali, Alex Widmer

**Affiliations:** ^1^ Institute of Integrative Biology ETH Zurich Zürich 8092 Switzerland; ^2^ Department of Ecology and Evolution University of Lausanne Lausanne 1015 Switzerland; ^3^ Faculty of Science Universiti Brunei Darussalam Gadong BE 1410 Brunei Darussalam

**Keywords:** Carnivorous plant, dioecy, molecular sexing, plant sex chromosome, privacy rarefaction, sex‐determination, sex‐specific loci

## Abstract

Species with separate sexes (dioecy) are a minority among flowering plants, but dioecy has evolved multiple times independently in their history. The sex‐determination system and sex‐linked genomic regions are currently identified in a limited number of dioecious plants only. Here, we study the sex‐determination system in a genus of dioecious plants that lack heteromorphic sex chromosomes and are not amenable to controlled breeding: *Nepenthes* pitcher plants. We genotyped wild populations of flowering males and females of three *Nepenthes* taxa using ddRAD‐seq and sequenced a male inflorescence transcriptome. We developed a statistical tool (privacy rarefaction) to distinguish true sex specificity from stochastic noise in read coverage of sequencing data from wild populations and identified male‐specific loci and XY‐patterned single nucleotide polymorphsims (SNPs) in all three *Nepenthes* taxa, suggesting the presence of homomorphic XY sex chromosomes. The male‐specific region of the Y chromosome showed little conservation among the three taxa, except for the essential pollen development gene *DYT1* that was confirmed as male specific by PCR in additional *Nepenthes* taxa. Hence, dioecy and part of the male‐specific region of the *Nepenthes* Y‐chromosomes likely have a single evolutionary origin.

**Impact Summary**

One of the most striking polymorphisms observed in organismal populations is the existence of male and female individuals. In contrast to animals, where this condition is common, plants are usually functional hermaphrodites. Some plants, however, are dioecious, that is, individuals are either of male or female sex. Dioecy has evolved hundreds of times independently in plants, which offers the potential for comparative studies of sex chromosome evolution and for investigating the genetic basis of transitions between hermaphroditism and dioecy (Charlesworth 2015). Yet empirical data to test hypotheses about why some species are dioecious and others hermaphroditic, and how such transitions are achieved, are lacking. Despite their potential, the sex‐determination mechanisms of most dioecious plants are not known, and few new species have been investigated since the seminal review by Westergaard (1958). Also, markers for molecular sexing have important applications in agriculture, horticulture, and conservation. Historically, the identification of sex‐determination systems was limited to species that can be bred in controlled conditions or have heteromorphic sex chromosomes. This is now changing with genome‐scale sequencing technology. Here, we investigated the sex‐determination system of carnivorous pitcher plants in the genus *Nepenthes*. We surveyed wild populations of three species by genotyping a large number of loci throughout their genomes. Because such data are noisy, we propose a solution to the common problem of distinguishing true signal from noise in presence–absence data by generating null distributions through permutations of the observed data. We discovered loci that occur only in males and reveal an XY sex chromosome system. One gene on the *Nepenthes* Y chromosome is particularly interesting, because it is presumably essential for pollen development and present only in males, and thus can be used to diagnose the sex of nonflowering plants

Although the majority of flowering plant species are functional hermaphrodites, plant sexual systems and sex‐determination mechanisms are highly diverse (Charlesworth 2002; Bachtrog et al. 2014). Only 5–6% of species have female and male flowers on separate individuals (dioecy), but the evolutionary transition to dioecy may have occurred as many as 800 times independently in angiosperms (Renner 2014). In contrast to outcrossing–selfing transitions due to loss of self‐incompatbility, for some of which the underlying genetic changes have recently been uncovered (e.g., Shimizu and Tsuchimatsu 2015), relatively little is known about the genes involved in transitions from hermaphroditism to dioecy and in sex determination in plants (Charlesworth 2016), although sex‐determining genes have been identified in three dioecious plant species: persimmon *(Diospyros lotus*, Akagi et al. 2014), *Asparagus officinalis* (Harkess et al. 2017; Murase et al. 2017), and kiwifruit (*Actinidia*, Akagi et al. 2018). The main hypotheses for the evolution of separate sexes in plants involve a combination of trade‐offs between the sex functions, plus disadvantage of inbreeding (Charlesworth and Charlesworth 1978).

Many dioecious plants have genetic sex determination, which may involve sex chromosomes. Sex chromosomes differ from autosomes by having suppressed meiotic recombination around the sex‐determining genes. These form a fully sex‐linked chromosomal region whose transmission is limited to one sex. When this sex is male, the system is referred to as male heterogamety (male genotype XY, female XX), and female heterogamety when the fully sex‐linked region is transmitted via females (male ZZ, female ZW). The sex‐specific, fully nonrecombining regions (male‐specific region of the Y, termed MSY, or female‐specific region of the W) show a number of special properties. First, an MSY may contain sequences that are absent from the X, and thus male specific (Y‐hemizygous, transmitted only from fathers to sons). As X and Y chromosomes are thought to evolve from a pair of autosomes, the gain of male‐specific sequences can be explained by several mechanisms, such as the rise of a new male‐determining mutation, or the translocation of a male‐determining cassette (Tennessen et al. 2018), or the localized expansion of repetitive sequences due to the lack of recombination. Second, over evolutionary time, the MSY may undergo genetic degeneration and lose functional genes that were initially shared with the X chromosome (Bachtrog 2013). Sex chromosomes have evolved independently many times in plants, and will therefore probably have diverse ages and levels of degeneration. Heteromorphic sex chromosomes have diverged sufficiently in size or structure to be distinguished optically with a microscope, whereas homomorphic sex chromosomes may have more subtle differences that can be detected only by molecular genetic methods. Despite their great potential for comparative studies, few plant sex chromosomes have been studied in detail (Ming et al. 2011; Harkess and Leebens‐Mack 2017; Muyle et al. 2017). Knowledge of sex‐determination systems and the identification of fully sex‐linked genetic markers are important for molecular sexing of juveniles or nonflowering adults in agriculture, breeding, and conservation.

Cytogenetics and linkage analysis in families are established methods to study sex determination and discover sex linkage of genes (Charlesworth and Mank 2010). However, these strategies fail in many dioecious organisms because their karyotypes are homomorphic (Filatov 2015), or because controlled breeding is difficult, since many dioecious plants are woody and reproduce only after many years (Renner and Ricklefs 1995). Several next‐generation sequencing techniques have now greatly increased knowledge about sex‐linked genes (reviewed by Muyle et al. 2017). However, they require either prior knowledge of heterogamety, controlled breeding, or whole‐genome sequencing, which remains expensive and time consuming. An alternative class of strategies uses population polymorphism to infer sex linkage of loci (reviewed by Muyle et al. 2017), even without pedigrees. These strategies can potentially allow sex‐linked regions to be discovered by cheaper reduced‐representation sequencing (RRS) methods such as RAD‐seq (Baird et al. 2008; Elshire et al. 2011; Peterson et al. 2012), although the gained information will remain incomplete, because typically only a few percent of a genome is covered. Nevertheless, the discovery of sex‐linked markers by RRS has been successful in organisms such as Crustaceans (Carmichael et al. 2013), *Anolis* lizards (Gamble and Zarkower 2014), geckos (Gamble et al. 2015a), and frogs (Brelsford et al. 2017; Jeffries et al. 2018).

A major problem faced by approaches that use population polymorphism to infer sex‐linkage, and sex specificity in particular, is error in the measurement of locus presence and absence (Text S4). Presence–absence error has long been recognized as a problem in fragment length genotyping methods, but it is exacerbated in RRS data, in which missing loci occur in a highly stochastic manner (Mastretta‐Yanes et al. 2015; Bresadola et al. 2019), and can make sex‐specific sequences appear in both sexes (Bewick et al. 2013; Gamble and Zarkower 2014; Heikrujam et al. 2015; Brelsford et al. 2017), and probably represent false positive results. One suggested solution to reduce the number of false positives is to compare increasing numbers of males and females (Gamble and Zarkower 2014; Gamble et al. 2015b). Unfortunately, in RAD data the number of shared loci decreases with increasing sample numbers (Mastretta‐Yanes et al. 2015). Beyond a number that is unpredictable and specific to each dataset, true sex‐specific loci may be missed because they are too rarely sequenced.

We developed a statistical procedure to deal with this problem, and applied it to characterize the sex‐determination system of *Nepenthes* pitcher plants. *Nepenthes* (Nepenthaceae, Caryophyllales) includes at least 160 species of perennial vines and shrubs occurring mostly in Southeast Asia (Cheek and Jebb 2001; Clarke et al. 2018). They are carnivorous plants that supplement their nutrition by killing and digesting animals in their modified pitcher leaves (Juniper et al. 1989; Moran and Clarke 2010; Pavlovič and Saganová 2015). All *Nepenthes* are dioecious, whereas close relatives (families Ancistrocladaceae, Dioncophyllaceae, Droseraceae, and Drosophyllaceae; Cuénoud et al. 2002; Renner and Specht 2011; Walker et al. 2017) are hermaphroditic. The individual male and female flowers (Fig. 1) are readily recognized because reproductive organs of the other sex abort early in development (Subramanyam and Narayana 1971). We hypothesized that sex in *Nepenthes* has a genetic basis, or is determined during early life stages, because there are no reports of sexual plasticity (Clarke 2001), or functional hermaphroditism. *Nepenthes* karyotypes (2*n* = 80, Heubl and Wistuba 1997) do not suggest heteromorphic sex chromosomes.

**Figure 1:**
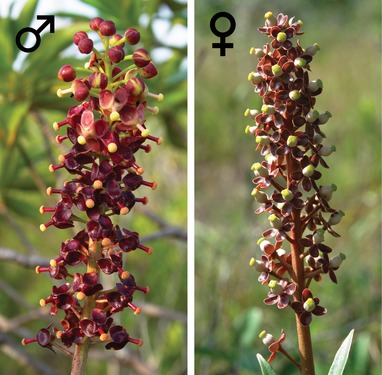
Sexual dimorphism in *Nepenthes* inflorescences. Left: male inflorescence of *N. rafflesiana s.l*. Right: Female inflorescence of *N. mirabilis* var. *globosa*. Photos: M. Scharmann

Here, we investigated the previously undescribed sex‐determination system of multiple *Nepenthes* species. Because controlled breeding of these slow growing plants faces many challenges, we sampled wild populations. We used *Silene latifolia* to test our method, as this species has well‐studied heteromorphic sex chromosomes. Specifically, we asked the following questions: (1) Are there sex‐linked loci in *Nepenthes*? (2) Are the same sex‐linked loci shared among different *Nepenthes* species? (3) Which expressed genes are sex linked? We discovered fully male‐specific and XY‐patterned loci and developed a molecular sexing assay for *Nepenthes*. The identified markers include two candidate sex‐determination genes, and these suggest that part of the Y chromosome is ancestral in this genus.

## Methods

### SAMPLING, ddRAD‐seq, AND GENOTYPING

Natural populations of *Nepenthes* were sampled in Brunei Darussalam (Borneo), Singapore, and the Seychelles. Fresh leaf material was stored in a nucleic acid preserving buffer (Camacho‐Sanchez et al. 2013). The sexes of *Nepenthes* plants were recorded from fresh or dry inflorescences. Scans for sex‐linked loci were conducted separately on three *Nepenthes* taxa: *N. pervillei* Blume, *N. gracilis* Korth., and *N. rafflesiana* sensu lato (Table 1). We extracted DNA from leaves using silica column kits (Nucleospin Plant II; Macherey Nagel, Düren, Germany) and prepared sequencing libraries following the ddRAD‐seq protocol (Peterson et al. 2012) using the enzymes *Eco*RI and *Taq*I. Library pools (84 or 96‐plex) were sequenced on an Illumina HiSeq 2500. Bioinformatic data filtering, *de novo* assembly of reference contigs (“RAD‐tags,” very short contigs with a mean length of c. 96 bases), read mapping, genotype calling, and quality filtering followed a modified dDocent pipeline (Puritz et al. 2014) and code is deposited at https://github.com/mscharmann.

**Table 1:** Sample sizes and origin for the taxa sequenced in this study

Taxon	Sampling location	Number of males	Number of females	Sequencing method
*Nepenthes pervillei* Blume	Seychelles, Mahé	28	22	ddRAD‐seq (Peterson et al. 2012)
*Nepenthes gracilis* Korth.	Brunei Darussalam, Borneo	10	10	ddRAD‐seq (Peterson et al. 2012)
*Nepenthes rafflesiana* sensu lato, here defined as:		39	22	ddRAD‐seq (Peterson et al. 2012)
*Nepenthes rafflesiana* “typical form” (Clarke 1992, 1997)	Brunei Darussalam, Borneo	13	7	
*Nepenthes rafflesiana* “giant form”(Clarke 1992, 1997)	Brunei Darussalam, Borneo	5	3	
*Nepenthes rafflesiana* Jack	Singapore	10	4	
*Nepenthes hemsleyana* Macfarl.	Brunei Darussalam, Borneo	11	8	
*Nepenthes khasiana* Hook.f.	cultivated/artificially prop.	1	–	RNA‐seq
*Silene latifolia* Poiret	Switzerland	27	32	GBS (Elshire et al. 2011)

The exploration of sex‐specific markers in *N. rafflesiana s.l*. followed an iterative strategy with two rounds of sexing, genotyping, bioinformatic analysis, and PCR validation (Text S1). To increase the phylogenetic range of our study and to validate molecular sexing, we also included individuals of known sex for additional species (Table S2‐1in Text S2). To validate our method for detection of sex‐specific loci, we also genotyped populations of a species with a well‐known, heteromorphic XY sex‐determination system, *S. latifolia* Poiret, using a single‐digest GBS protocol (Elshire et al. 2011). Details of the *Silene* samples and genotyping are provided in Text S3.

### DETECTION OF SEX‐LINKAGE

We distinguished between sex‐linked loci showing sex‐specificity, meaning without homology between the two sex chromosomes (e.g., Y‐linked loci whose fully X‐linked copy is absent or undetectable by our methods), and loci that are present on both sex chromosomes but whose allele frequencies diverged between the sex chromosomes, called ZW‐ or XY‐patterned variants (Gammerdinger and Kocher 2018). We define sex‐specific loci as contigs to which sequencing reads can be aligned from only one of the sexes (presence–absence polymorphism). However, this need not reflect true presence–absence, because observed absence may be due to methodological factors (deliberate or random) specific to each dataset. Imperfect detection may be caused by many underlying factors, including the number of male and female individuals investigated, their genetic relatedness, the species’ genome size and structure, the library preparation method, sequencing depth, and bioinformatic processing. Consider, for example, a particular genetic marker scored “present” in nine out of ten males and “absent” in five out of five females. True complete male specificity cannot be distinguished from technical artifacts due to random variation, for example, in sequencing depth or sampling bias. To improve the inference of sex specificity and facilitate comparisons of datasets from different species, sampling schemes, and sequencing runs, we used a resampling strategy that evaluates the biological signal among dataset‐specific artifacts and uncertainties. The procedure is implemented in python and named “privacy rarefaction” (https://github.com/mscharmann). Further details, including a performance analysis with simulations, are presented in Texts S4 and S5.

We tested for completely or partially XY‐ or ZW‐patterned SNPs, that is, ones with different allele and heterozygote frequencies in the two sexes, using genotypes and PLINK version 1.07 (Purcell et al. 2007). Associations between biallelic SNPs and sex were analyzed by chi‐squared tests, and candidate SNPs were accepted as sex associated at a false discovery rate of 5% (Benjamini and Hochberg 1995), and then classified as XY‐patterned if males were predominantly heterozygous, or as ZW‐patterned if females were predominantly heterozygous. To perform this test on a reasonable number of SNPs, we allowed data from up to 25% of individuals to be missing per SNP.

### POPULATION GENETICS OF CANDIDATE SEX‐LINKED LOCI

We tested whether linkage disequilibrium (LD), that is, the nonrandom association of alleles at separate loci, differed in male samples between sex‐linked regions and the genomic background (represented by 15,000 randomly selected pairs of nonsex‐linked SNPs). Stringent quality filters were applied: singleton SNPs were removed, SNPs in male‐specific contigs were excluded if any heterozygous genotypes were called in the males (because Y‐hemizygosity implies that heterozygosity is not true, so these were probably paralogous sequences), and for non‐sex‐linked contigs any excessively heterozygous SNPs (Hardy–Weinberg test with significance level 5%) were excluded. Excessively heterozygous SNPs were retained for XY‐patterned contigs. LD (*r* ^2^) was calculated exclusively for SNPs from different contigs using PLINK. The same contrasts were made for nucleotide diversity π, which was averaged per contig for all SNP‐containing contigs (including singleton SNPs) in VCFtools version 0.1.15 (Danecek et al. 2011) and for contigs without SNPs taken directly from bam alignments. The same filters were applied to both data (minimum read depth 3, maximum read depth 75, and minimum population presence 0.75). The significance of differences of the means was evaluated by randomization (10,000 rounds of re‐sampling without replacement from the two groups, randomizing group membership).

### COMPARISON OF CANDIDATE LOCI TO A MALE INFLORESCENCE TRANSCRIPTOME

We sequenced and assembled the transcriptome of a single developing male inflorescence of *Nepenthes khasiana* Hook.f. (Text S6) to identify and annotate sex‐linked candidate loci. Fresh inflorescences of the species used for ddRAD‐seq were not available in cultivation. The transcriptome was searched (a) by BLAST for similarity to candidate contigs (thresholds ≥90 aligned bases and ≥75% identity) and (b) by repeating privacy rarefaction with ddRAD‐seq reads directly mapped to the transcriptome rather than the RAD‐tag reference (bwa mem; Li 2013; retaining multiple mappings).

Candidate transcripts from both approaches were annotated by BLAST search against the NCBI Genbank nucleotide collection (November 7, 2016 version) and the nonredundant protein collection (March 26, 2016 version). Transposable elements (TEs) were detected using RepeatMasker 4.0.6 (Smit et al. 2013) version 20150807 (eukaryota). Proteins with at least 50 amino acids were predicted by TransDecoder (Trinity package) and annotated against nr, UniProt Swiss‐Prot (August 17, 2016 version), and *Arabidopsis thaliana* proteins in UniProtKB (April 3, 2016 version). PFAM domains were detected using hmmer 3.1b1 (Eddy et al. 2016), accepting hits at *e*‐value ≤10^−5^.

### PCR VALIDATION

Candidate sex‐specific contigs were chosen for PCR validation based on a ranking of the privacy rarefaction results (using the highest stringency level reached and the bootstrap support), taxonomic overlap, and the quality of annotation of matching transcripts. PCR primers were designed in Geneious R6 (Biomatters Ltd., Auckland, New Zealand), and tested according to the protocol described in Text S2.

## Results

### SEX‐LINKED LOCI

**Figure 2:**
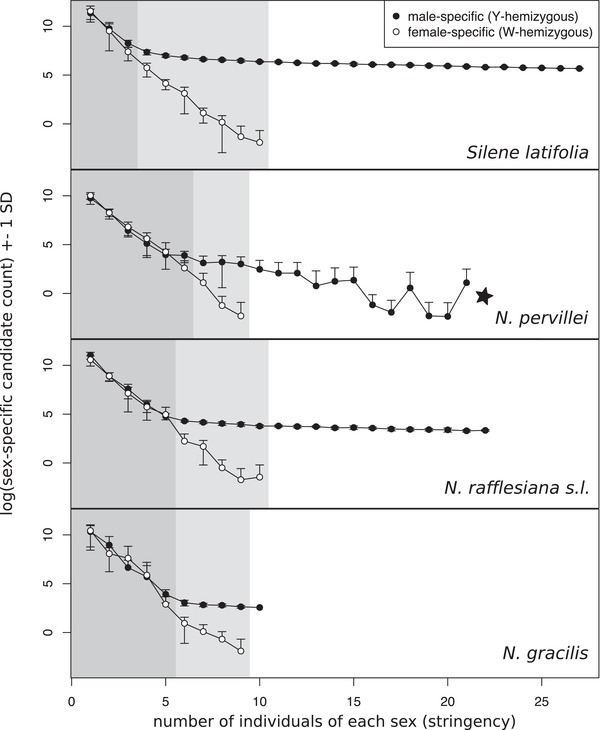
Evidence for male‐specific loci and XY sex‐determination systems in *Silene latifolia* and three *Nepenthes* spp. (privacy rarefaction curves). Shown are counts of sex‐specific contigs (*y*‐axis) as a function of the number of individuals of each sex sampled to score sex specificity (*x*‐axis, stringency). Sex‐specific contigs are defined as those to which sequencing reads from only one sex can be aligned. Dots represent averages, and whiskers one standard deviation of 200 bootstrapped combinations of males and females. Note natural log‐scale of *y*‐axis and hence undefined zero and negative values in the SD ranges. The background shading of the plots indicates three relevant zones that are directly informative on the sex‐determination system: a dark gray zone (low stringency) indicates no difference between the sexes, the light gray zone (intermediate stringency) highlights where significant differences between sexes are found, and white background (highest stringency) shows the biologically plausible zone where sex‐specific markers are obtained in only one sex. Male‐specific candidates were found in all species up to the maximum possible stringency (the minimum number of male individuals and female individuals), except in *N. pervillei* (asterisk).

We first searched for sex‐specific contigs in the illustrative example of *S. latifolia* GBS data using privacy rarefaction. When small numbers of individuals of each sex were analyzed, the procedure yielded similar numbers of male‐ and female‐specific candidates (Table S8; Fig. 2, top, dark gray zone), which decreased monotonically with greater numbers of individuals, as expected. When the numbers of males and females analyzed were increased, a clear signal of male heterogamety emerged. With four or more individuals of each sex analyzed, the proportion of male‐specific candidate loci increased and finally became significantly greater than the number of female‐specific candidates (Fig. 2, top, light gray zone). At 11 and more individuals of each sex analyzed, the number of female‐specific candidates dropped to zero, whereas the number of male‐specific candidates remained high (Fig. 2, top, white zone). Hence, these curves correctly diagnosed an XY‐system for *S. latifolia*, and rejected a ZW‐system. Due to this characteristic drop‐out of false‐positives, we also refer to the numbers of analyzed individuals of each sex as “privacy rarefaction stringency.” Some of the herein identified male‐specific *S. latifolia* contigs were previously reported to be sex‐linked (Text S3, Table S11).

Qualitatively consistent signatures of male‐specific contigs were detected independently in *N. pervillei*, *N. gracilis*, and *N. rafflesiana s.l*. (Fig. 2, Table S8). The proportion of male‐specific loci among all loci was, however, about 10‐fold lower in *Nepenthes* than *S. latifolia*. Estimates based on subsamples of 10 males and 10 females were only 0.02% for *N. pervillei* (11.8/51,002), 0.11% for *N. rafflesiana s.l*. (43.7/40,508.7), and 0.06% for *N. gracilis* (13/22,789), versus 1.52% for *S. latifolia* (586.4/38,455).

In *N. pervillei* and *N. rafflesiana s.l*., as well as in *S. latifolia*, we also detected XY‐patterned SNPs, but not in *N. gracilis*, while none of the species yielded any ZW‐patterned SNPs. The XY‐patterned SNPs for *S. latifolia* recovered 289 contigs already known to be sex linked in that species, which, however, represent only a small fraction of the known and theoretically expected *S. latifolia* sex‐linked sequences (c. 1/7 of the genome; Text S3, Table S11). This low power was expected for a sequencing strategy that targets only a small subset of the genome. Almost all XY‐patterned SNPs had an allele frequency close to 0.5 and near‐complete heterozygosity in males, but were homozygous in females (Table S9). The proportions of XY‐patterned SNPs were also much lower in *Nepenthes* than in *S. latifolia* (2376/149,311 = 1.6%, similar to the proportion of male‐specific loci): the estimates were, respectively, 0.25% and 0.017% for *N. pervillei* (97/38,783) and *N. rafflesiana s.l*. (37/222,188).

### POPULATION GENETICS OF SEX‐LINKED LOCI

Fully Y‐linked loci experience no recombination, which should lead to increased population LD between different male‐specific contigs. All three testable male‐specific contigs of *N. pervillei* showed perfect LD (*r* ^2^ = 1, which was c. 0.7 units higher than the genomic background; *N* = 3 SNP pairs, *P* = 0.06). Likewise, the male‐specific contigs of *N. rafflesiana s.l*. (containing 82 SNP pairs) had an *r* ^2^ that was on average c. 0.4 above the genomic mean (*P* < 10^−5^); the median value was complete LD (*r* ^2^ = 1). Among contigs with XY‐patterned SNPs, *r* ^2^ was 0.15 higher than the background mean in *N. pervillei* (*P* = 10^−5^, *N* = 147), but was no different from the mean in *N. rafflesiana s.l*. (*P* = 0.56, *N* = 11). Our observation of some low LD values between sex‐specific contigs is likely due to the discreteness of allele frequency estimates from small sample sizes, and the allele frequency dependence of LD metrics, whose maximum possible value is frequently much less than 1.0 (VanLiere and Rosenberg 2008).

**Figure 3:**
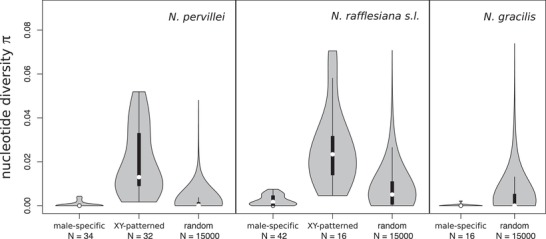
Mean per‐site nucleotide diversity π of contigs in male *Nepenthes* of three taxa for male‐specific, XY‐patterned, and random nonsex‐linked contigs. All contigs mapping 3–75 reads in ≥75% of males per population were included. The same sets of individuals are considered in each category. No XY‐ or ZW‐patterned contigs were found in *N. gracilis*. Median = white dot, box = 25–75% quartiles, whiskers = 1.5^*^interquartile range, violin = estimated kernel density.

The mean nucleotide diversity π in male‐specific (putatively Y‐linked) contigs tended to be lower than the genomic background in all three taxa (Fig. 3), consistent with theoretical expectations for Y‐specific loci, whose effective population size is only 1/4 of that of autosomal genes, and can be reduced much further by genetic hitchhiking and high variance among males in the number of sired offspring (Wilson Sayres et al. 2014). This difference was significant for *N. rafflesiana s.l*. (*P* < 10^−3^), but not for *N. pervillei* or *N. gracilis* (*P* = 0.13 and *P* = 0.055, respectively). In contrast, mean π of males in contigs with XY‐patterned SNPs was higher than the genomic mean for both *N. pervillei* (*P* = 10^−5^, Fig. 3) and *N. rafflesiana s.l*. (*P* = 0.002; Fig. 3), a consequence of high male heterozygosity in the XY‐patterned SNPs.

### SHARED CANDIDATE LOCI BETWEEN SPECIES, FUNCTIONAL ANNOTATIONS, AND PCR VALIDATION

Six candidate sex‐specific contigs were found at privacy rarefaction stringency level ≥5 that were shared between *N. gracilis* and *N. rafflesiana s.l*. No sex‐specific candidates were shared between *N. pervillei* and the other species (Table S10). There was no overlap in XY‐patterned SNPs between the *Nepenthes* species, and no direct overlap between male‐specific contigs and XY‐patterned ones. However, one male‐specific contig of *N. gracilis* and one XY‐patterned contig of *N. rafflesiana s.l*. both matched (full length alignment, *e*‐value ≤1 × 10^–19^) to the same inflorescence transcript containing a DUF4283 (domain of unknown function, http://pfam.xfam.org/family/PF14111, November 9, 2016).

One male‐specific contig of *N. pervillei* aligned to the transcript of a bHLH transcription factor, and the best matches in all accessed databases were consistently to predicted orthologs of the *Arabidopsis* gene *DYSFUNCTIONAL TAPETUM1* (*DYT1*). A further XY‐patterned contig of *N. pervillei* matched a transcript annotated as *A. thaliana* *SEPALLATA‐1 (SEP1)*, which aligned to the predicted 3′‐UTR of the putative *N. pervillei SEP1*‐ortholog, and contained two SNPs that were both homozygous in 95% of females and heterozygous in 96% of males. No estimate of *SEP1* X–Y divergence was possible because the male inflorescence transcriptome reads were not heterozygous. In *N. gracilis*, a male‐specific contig matched a long transcript similar to a mitochondrial NADH‐ubiquinone oxidoreductase from *Beta vulgaris* (Swiss‐Prot). This finding was unexpected and may represent either an unspecific match of the short (96 bp) contig to the inflorescence transcript, or else a cyto‐nuclear transfer to the sex chromosomes. The occurrence of organellar genes on plant sex chromosomes has been documented in other species (Steflova et al. 2013).

All other candidate loci either included traces of TEs, or had no known sequence motifs (Table S10). In particular, of the 38 sex‐linked inflorescence transcripts identified here (by 41 matching sex‐linked contigs), 34 (89%) could not be annotated, or contained TEs. TEs were commoner than in nonsex‐linked transcripts (*χ* ^2^_df = 1_ = 5.2, *P* = 0.02). Nine out of 13 sex‐linked TE‐transcripts annotated as *gypsy*‐like retrotransposons, a significant overrepresentation relative to nonsex‐linked TE transcripts (*χ* ^2^_df = 1_ = 8.85, *P* = 0.003).

Complementary to the sex‐specificity scan on the ddRAD‐de novo reference, we repeated privacy rarefaction by directly mapping the ddRAD reads to the male inflorescence transcriptome to find further annotated sex‐linked genes. This identified seven transcripts (Table S10) that map male‐specific regions of the genomes. We recorded only high‐confidence male‐specific candidate transcripts present in at least four males and absent in at least four females, and bootstrap support greater 0.5 in at least one species. No female‐specific transcripts (false positives) reached these support levels. A single transcript was male‐specific in *N. rafflesiana s.l*. but could not be annotated. Four close transcript “isoforms” (contigs that share sequence but differ slightly in structure, as assembled by Trinity; Haas et al. 2013) were male specific in both *N. gracilis* and *N. rafflesiana s.l*., but they lacked similarity to any known motif except for one isoform similar to a Jockey‐1_Drh retrotransposon. However, two transcripts were male specific in both *N. pervillei* and *N. rafflesiana s.l*., and one of these also matched a *N. pervillei* male‐specific contig (see above). These two transcripts appear to be close isoforms (putative intron presence–absence), and both annotated as *DYT1* (see above).

We tested by PCR whether the putative *DYT1*‐ortholog is male specific in a broad range of *Nepenthes* species. A single PCR product of approximately 290 bp length was observed exclusively and consistently in phenotypically sexed male *Nepenthes* but never in females (Text S2). Multiple males and females were screened in eight taxa, and 1–2 individuals from 14 further taxa. Presence–absence of the PCR product was fully consistent with the phenotypic sex of all 56 individuals. Sanger sequencing of the PCR product confirmed the identity of the target region. Hence, this locus is male specific across a phylogenetically broad range of *Nepenthes* species and can be used for molecular sexing.

### PERFORMANCE OF THE RESAMPLING STRATEGY

We tested the performance of privacy rarefaction on simulated datasets resembling typical RAD‐seq experiments under a range of missing data levels, sampling schemes, and sizes of the sex‐specific region (Text S5). The procedure correctly identified the heterogametic sex in >90% of simulations when the proportion of sex‐specific contigs in the genome was at least one permil, and virtually all contigs classified as sex specific were true positives. A naive scoring method, in contrast, failed to detect the heterogametic sex in most scenarios because sex‐specific contigs appeared in both sexes, and it typically reported many false positives. However, most of the true sex‐specific contigs were not detected in simulated RAD‐seq data because of the missing data inherent to this sequencing method (low sensitivity). Nevertheless, the relative size of the sex‐specific regions was usually estimated to the true order of magnitude (Table S5‐1 in Text S5).

## Discussion

### THE *NEPENTHES* SEX‐DETERMINATION SYSTEM

Our findings reveal that sex determination has a genetic basis in *Nepenthes* and involves a nonrecombining region in males. *Nepenthes* karyotypes suggest that the sex chromosomes are homomorphic (Heubl and Wistuba 1997), consistent with the lower proportions of sex‐linked contigs in *Nepenthes* compared to *S. latifolia* with its large and heteromorphic Y‐chromosome. The proportions of male‐specific contigs allow us to hypothesize that the size of the MSY relative to the whole genome is about 10‐fold smaller in *Nepenthes* than in *S. latifolia*, and that within the genus, it is smallest in *N. pervillei*. However, we note that the characterization of sex chromosomes via reduced‐representation sequencing methods necessarily remains incomplete (Text S3), and very strict analyses, such as the resampling procedure we propose here, are required to avoid false inferences (Text S5). Furthermore, our sampling included several subpopulations in *S. latifolia* and *N. rafflesiana s.l*., which may have impeded the detection of deme‐specific sex‐linked loci.

**Figure 4:**
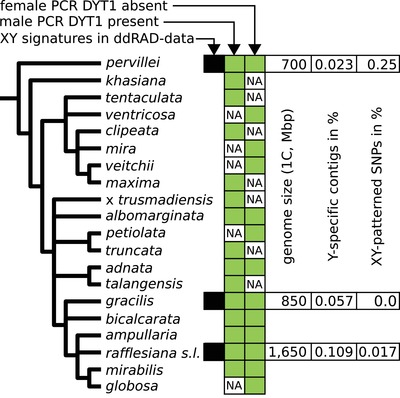
Summary of results on the sex‐determination system for *Nepenthes*, annotated on a plastid phylogeny (after Meimberg and Heubl 2006). The crown of the genus is c. 17.7 (CI 11.0‐24.3) million year old (Text S7). It constrains the minimum age at which dioecy evolved and *DYT1* became a male‐specific gene. NA = not available/not tested. Genome sizes were quantified by flow cytometry. The proportion of *Y*‐specific contigs is given at 10 individuals of each sex (stringency). *Nepenthes rafflesiana* s.l. contains several entities, for which the PCRs were conducted separately.

The MSY of *Nepenthes* appears to contain a “core region” that is conserved throughout the genus. The *DYT1* gene was male specific in both *N. pervillei* and *N. rafflesiana s.l*., and part of it was consistently PCR amplified in known males but never in females of 22 *Nepenthes* species (Text S2), representing all major clades (Fig. 4; Mullins 2000; Meimberg et al. 2001; Meimberg and Heubl 2006; Scharmann et al. unpubl. data). The shared MSY locus therefore suggests a single origin of dioecy in *Nepenthes* that most likely predates the most recent common ancestor of extant *Nepenthes* at 17.7 (CI 11.0–24.3) million years ago but followed the split between Nepenthaceae and hermaphroditic Droseraceae at least 44.2 million years ago (average 71.1 with CI 44.2–98.0; Text S7). However, the age of the shared MSY core does not necessarily reflect the age of the sex chromosomes: their identity could have changed over time and may also differ between *Nepenthes* species because the ancestral MSY could have been translocated to other chromosomes in a process called “sex‐chromosome turnover” (Blaser et al. 2014; Jeffries et al. 2018; Tennessen et al. 2018). Nevertheless, the *Nepenthes* MSY core is probably older than the heteromorphic *S. latifolia* sex chromosomes (∼11 million years, Krasovec et al. 2018). These alternatives can be explored in future comparative whole genome sequencing or mapping studies.

During the radiation of *Nepenthes*, the MSY has diverged between species, as is expected over such long divergence times, particularly for noncoding sequences. Only six out of 135 male‐specific contigs were shared between *N. rafflesiana s.l*. and *N. gracilis*, and none were shared with the more distant *N. pervillei* (Fig. 4). Male‐specific loci shared between *N. pervillei* and *N. rafflesiana s.l*. were only recovered with the help of longer, transcriptome contigs to align ddRAD reads. Absence of shared male‐specific contigs should not, therefore, be interpreted as evidence for independent origins of sex chromosomes, but rather reflects sequence divergence between species. Further evidence for a common origin followed by interspecific divergence is found in a DUF4283 transcript, which is male specific in *N. gracilis* but XY‐patterned in *N. rafflesiana s.l*., suggesting X and MSY alleles (i.e., gametologs) have lost sequence similarity in the former but not in the latter species.

### NONCODING DNA AND SPECIAL SIGNIFICANCE OF *DYT1* AND *SEP1*

Nonrecombining regions of sex chromosomes accumulate repetitive, noncoding sequences and TEs in species with both heteromorphic or largely homomorphic sex chromosomes (Čermák et al. 2008; Wang et al. 2012). In *Nepenthes*, most sex‐linked genomic regions detected by our approach were noncoding sequences and TEs and only a few genes with putative developmental functions were identified. Of these, a *Nepenthes* homolog of *DYT1* appears to be located in the MSY of all *Nepenthes* species. *DYT1* is essential for tapetum development and thus pollen fertility in *A. thaliana* (Zhang et al. 2006), rice (Jung et al. 2005; Wilson and Zhang 2009; Cai et al. 2015), and tomato (Jeong et al. 2014). Given this gene's functional conservation in these distantly related Angiosperms, we speculate that its function is the same in *Nepenthes*, and future ork could validate this hypothesis, for example, via transient transformation of *Nepenthes* (Miguel et al. 2019). Our analysis suggests that *DYT1* is absent from *Nepenthes* females and must thus be absent from the X chromosome. Such a deletion of *DYT1* from the X chromosome would constitute a recessive male‐sterility mutation, as required early in the evolution of dioecy for the transition from a hermaphroditic to a gynodioecious mating system (Charlesworth and Charlesworth 1978). It is notable that in *Arabidopsis*, *DYT1* directly regulates the expression of TDF1 (Gu et al. 2014), a gene that in dioecious asparagus is essential for male fertility and, like *DYT1* in *Nepenthes*, is located in the MSY (Harkess et al. 2017; Murase et al. 2017). Apparently, this pollen development pathway was involved twice independently in the evolution of angiosperm XY chromosomes, and possibly in the transition to dioecy.

The second *Nepenthes* gene of interest is a homolog of the homeotic MADS box gene *SEP1*, an early‐acting regulator of floral organ identity in *A. thaliana* (Pelaz et al. 2000), which was XY‐patterned in *N. pervillei*. Two *SEP1*‐linked SNPs were heterozygous in 27 of 28 males, whereas 21 of 22 females were homozygous, consistent with the existence of strongly X‐ and Y‐linked copies. If *SEP* homologs in *Nepenthes* are involved in the determination of floral organ identity (as in *A. thaliana*, Theißen et al. 2016), the sex‐linked *Nepenthes* *SEP1* homolog could be involved in unisexual flower development. In particular, sequence differences between the *Nepenthes* *SEP1* X‐ and Y‐linked copies might modify their functions such that they suppress the development of either carpels or stamens. In *S. latifolia*, however, *SEP1* homologs are not directly involved in sex determination and are not located on the sex chromosomes (Matsunaga et al. 2004).

The possible roles of *DYT1* and *SEP1* in the origin of dioecy in *Nepenthes* require further attention. Even if these are not primary sex‐determining genes in extant *Nepenthes*, they might have been under sexually antagonistic selection during the evolution of dioecy because loss of function of *DYT1* or *SEP1* alleles might abort nonfunctional organs at early developmental stages, thus saving resources. The fully unisexual morphology of extant *Nepenthes* flowers (Subramanyam and Narayana 1971) implies further developmental genetic differences between males and females.

## Conclusion

This study reports the discovery of an XY sex‐determination system in dioecious pitcher plants (*Nepenthes* spp). The sex chromosomes are homomorphic with a small Y‐specific region, which has a relatively old core that is shared between distinct species. The nonrecombining region is enriched for noncoding sequences and TEs, but also contains several expressed genes with putative developmental functions.

Associate Editor: S. Wright

## Supporting information


**Text S1**. Preliminary molecular sexing assay for *Nepenthes rafflesiana s.l*.
**Text S2**. A molecular sexing assay for the genus *Nepenthes*.
**Text S3**. Analyses of sex‐linkage in *Silene latifolia*.
**Text S4**. Privacy rarefaction.
**Text S5**. Performance analysis of privacy rarefaction on simulated RAD data.
**Text S6**. Male inflorescence transcriptome of *N. khasiana*.
**Text S7**. Phylogenetic dating of *Nepenthes*.

**Table S8**. Result tables of privacy rarefaction for three *Nepenthes* and *Silene*.

**Table S9**. Result tables of association tests for SNPs with sex and heterozygosities.

**Table S10**. Summary table of sex‐linked loci including overlap between species and annotations for RAD‐tag and transcriptome references.

**Table S11**. Data table for *S. latifolia* sex‐linkage comparison of our results to previous studies.
