# Supplementary material for: Sex is determined by XY chromosomes across the radiation of dioecious Nepenthes pitcher plants
Source: Evol Lett. 2019 Oct 1;3(6):586–97. doi: 10.1002/evl3.142 (PMC6906984; doi:10.1002/evl3.142)
Supplement: Supplementary file 1 — Text S1. Preliminary molecular sexing assay for Nepenthes rafflesiana s.l. Text S2. A molecular sexing assay for the genus Nepenthes. Text S3. Analyses of sex‐linkage in Silene latifolia. Text S4. Privacy rarefaction. Text S5. Performance analysis of privacy rarefaction on simulated RAD data. Text S6. Male inflorescence transcriptome of N. khasiana. Text S7. Phylogenetic dating of Nepenthes. [file EVL3-3-586-s001.doc]

**Supporting Information S1 - S7 for**

**Scharmann, M., Grafe, T.U., Metali, F. & Widmer, A. (2019). Sex is determined by XY chromosomes across the radiation of dioecious *Nepenthes* pitcher plants. Evolution Letters.**

**Text S1** **Preliminary molecular sexing assay for *Nepenthes rafflesiana s.l.***

Here we describe the preliminary first round of sexing, genotyping, bioinformatic analysis, and PCR-validation for *N. rafflesiana s.l.* and how these results were used in subsequent analyses reported in the main text. An initial sequencing library contained phenotypically sexed individuals of *Nepenthes rafflesiana* "typical form" (15 males, 15 females) and *N. gracilis* (5 males, 5 females) from Borneo. Collection and DNA extraction are detailed in the main text. We commissioned the Genomic Diversity Facility (Cornell University, Ithaca, NY, USA) with library construction and sequencing, following the GBS protocol (Elshire *et al.* 2011). After optimisation, the restriction enzyme Sfb1 was chosen and the library was sequenced for 100-bp single-end reads in two Illumina HiSeq lanes.

At this early stage, we employed a simpler version of the resampling approach to detect sex-specific loci, using the Stacks pipeline (Catchen *et al.* 2013) instead of the dDocent approach (Puritz *et al.* 2014) for genotyping. The populations module of Stacks was iterated over different combinations of real and permuted males and females, thereby revealing numbers and identities of likely sex-specific loci following the same logic as described in the main text ("privacy rarefaction"). This attempt was successful in *N. rafflesiana* "typical form" (Borneo), but failed to identify any sex-specific loci in *N. gracilis*. We took the top 10 best candidate loci for *N. rafflesiana* "typical form" (Borneo) and designed PCR primers for validation of sex-specificity (PCR conditions as described in the main text). Two of these loci amplified from males exclusively (private gel band at expected size), as verified in all males that were used for the genotyping and several further samples that had not been used previously. The same markers also amplified specifically from known males but not females of *N. hemsleyana* and *N. rafflesiana* "giant form" (Borneo). However, these markers were not sex-specific for any other tested species (*N. ampullaria, N. bicalcarata, N. gracilis, N. mirabilis*). We consequently used these two markers to molecularly sex additional individuals of *N. rafflesiana* "typical form" (Borneo) and *N. hemsleyana* that were included in full study and genotyped with the ddRAD-seq approach. To conclude, the sexes of most *N. hemsleyana* and several of the *N. rafflesiana* "typical form" (Borneo) individuals were determined not on the phenotype but based on molecular markers developed from a pilot GBS dataset.

**Text S2 A molecular sexing assay for the genus *Nepenthes***

Based on the evidence for male-specific genomic regions (non-recombing Y-chromosomal region), we developed an assay to sex *Nepenthes* molecularly. Here we test it with phenotypically sexed individuals from 22 different *Nepenthes* spp. (Text S2 Table S2-1). The assay is likely applicable to further *Nepenthes* spp., but we recommend to validate it using several phenotypically sexed individuals before application to a novel species.

S2 Table S2-1. *Nepenthes* spp. with phenotypically verified sex used for broader taxonomic validation of a male-specific PCR marker

| species | N_male | N_female | source |
| --- | --- | --- | --- |
| *adnata* | 1 | 1 | cultivated |
| *albomarginata* | 1 | 3 | wild populations, cultivated |
| *ampullaria* | 2 | 2 | wild populations |
| *bicalcarata* | 2 | 2 | wild populations |
| *clipeata* | 1 | 0 | cultivated |
| *gracilis* | 3 | 3 | wild populations, cultivated |
| *hemsleyana* | 2 | 2 | wild populations |
| *khasiana* | 1 | 0 | cultivated |
| *maxima* | 2 | 0 | cultivated |
| *mira* | 0 | 1 | cultivated |
| *mirabilis* | 3 | 3 | wild populations |
| *mirabilis var. globosa* | 0 | 1 | cultivated |
| *pervillei* | 3 | 3 | wild populations |
| *petiolata* | 0 | 1 | cultivated |
| *rafflesiana typical form* Borneo | 3 | 3 | wild populations |
| *singalana* | 1 | 0 | cultivated |
| *talangensis* | 1 | 0 | cultivated |
| *tentaculata* | 1 | 0 | cultivated |
| *truncata* | 1 | 0 | cultivated |
| *veitchii* | 0 | 1 | cultivated |
| *ventricosa* | 0 | 1 | cultivated |
| x *trusmadiensis* | 1 | 0 | cultivated |

*DNA extraction*

*Nepenthes* tissue contains strong PCR inhibitors, as simple extraction protocols without purification (as suggested by Hobza & Widmer 2008) did not yield amplification products. We thus used the silica-column kit NucleoSpin Plant II from Macherey Nagel (Düren, Germany). For optimal yield, the tissue was completely powdered before the lysation step. To achieve this, tissue was flash-frozen in liquid nitrogen and then crushed in disposable, folded paper envelopes using pliers. While still frozen, the resulting coarse powder was transferred to a 2 ml cryotube (Sarstedt No. 72.694.005, Screw Cap Micro Tube, 2 ml, PP, conical and skirted base) with three steel beads. On a shaker mill, cycles of shaking (up to 15 s) and flash-freezing in a liquid nitrogen bath were repeated until the material was a fine dust. Acceleration on the shaker mill was carefully adjusted to the maximum possible level that did not break the frozen cryotubes. Lysis buffer was added directly to the tissue dust without prior thawing. All other steps followed manufacturer instructions.

*PCR amplification of control and sex-specific sequences*

The assay involves four primers: one pair targets a male-specific region (within the putative *Nepenthes* ortholog of the *Arabidopisis thaliana* DYT1 gene, 25100_L96_2_F: 5'-AATTCACTGATTCGGATCACG-3'; 25100_L96_294_R: 5'-CGATCGCGTCGCAAAGTATG-3'), while the other targets a sequence that is common to both sexes (the mitochondrial *cox1* gene; IP53: 5'-GGAGGAGTTGATTTAGC-3'; cox1.6KR: 5'-AAGGCTGGAGGGCTTTGTAC-3'; Cho *et al.* 1998). As suggested by Hobza & Widmer (2008), the common target is used as an internal control of each reaction. It ensures that poor template quality or other technical issues are recognised as such, instead of confusion with true absence of the sex-specific target, i.e. the expectation for females. The two regions can also be amplified in separate reactions.

The reaction is performed in 15 µl volumes containing 2.5 mM MgCl_2_, 250 µM of each dNTP, 0.375 units of GoTaq DNA polymerase (Promega, Wisconsin, USA), 1x GoTaq Flexi buffer (Promega), 0.5 µM of each of the four primers, and 1 µl of template DNA (c. 5-20 ng/µl). After initial denaturation for 2 min at 95°C, 30 cycles are run with denaturation for 30s at 95°C, annealing for 30s at 50°C and extension for 1 min at 72°C, followed by a final extension step of 5 min at 72°C (Thermocycler, e.g. Labcycler, SensoQuest, Göttingen, Germany).

*Visualisation and scoring of PCR products*

PCR products were separated on 2% agarose gels and visualised by fluorescent dye (Text S2 Fig. S2-1). Successful assays contain at least one strong band at 600-700 bp length, corresponding to the control *cox1* fragment. The presence of a strong band at c. 290 bp characterises male individuals, while females do not display this band. Several other, weaker bands of different length may be present. These are likely unspecific products of the control primer pair, as we could never observe them when applying the sexing primer pair exclusively.


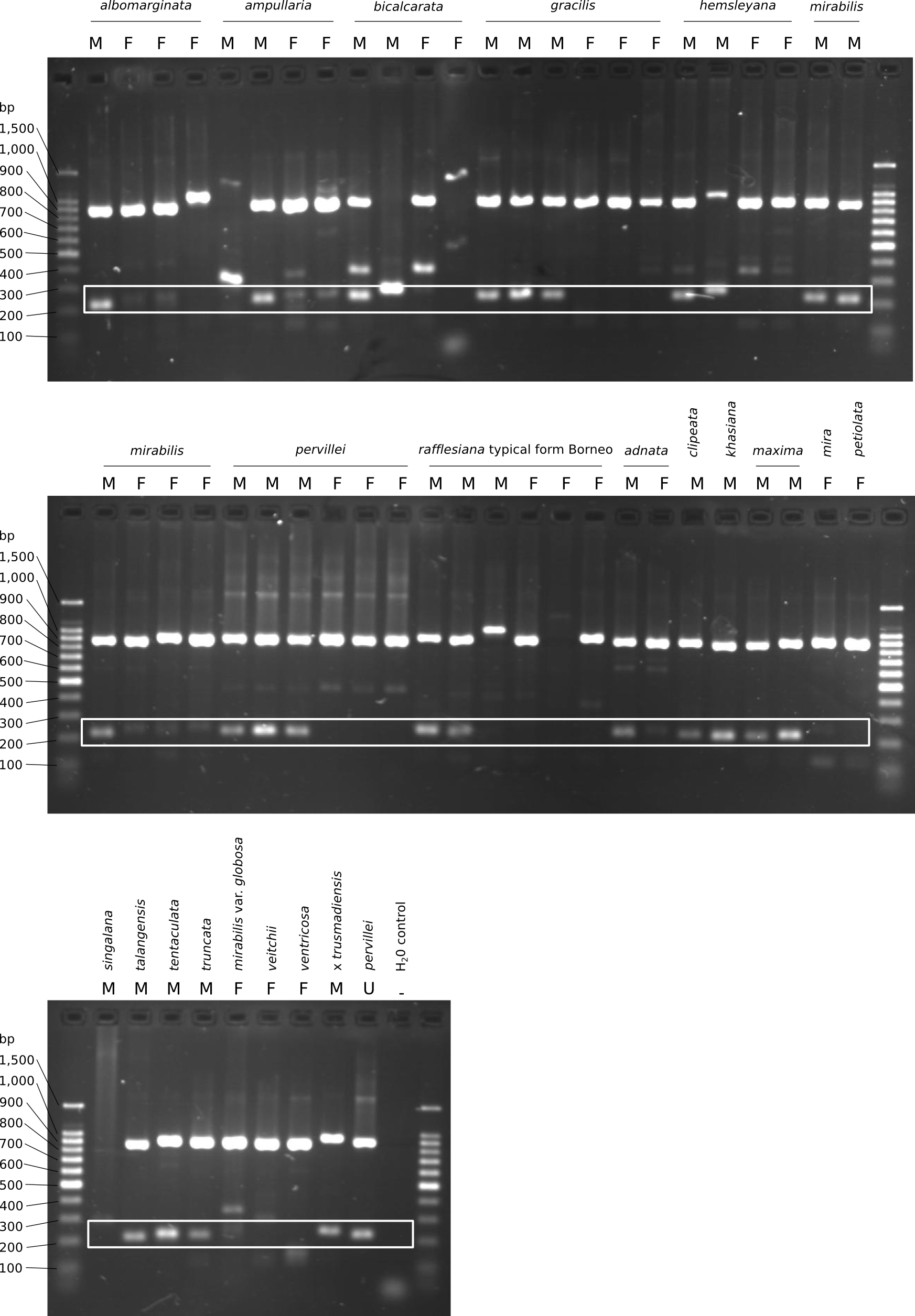


S2 Fig. S2-1. Images of agarose gels confirming molecular sexing of *Nepenthes* spp. listed in S2 Table S2-1. The section containing the male-specific band is highlighted with a white box.

**Text S3. Analyses of sex-linkage in *Silene latifolia***

*Methods*

Wild populations of *Silene latifolia* were sampled across Switzerland and leaves preserved by drying in silica gel. The phenotypic sex of individuals was recorded. After DNA extraction (Qiagen DNeasy Plant Mini Kit), library construction was comissioned at the Genomic Diversity Facility (Cornell University, Ithaca, NY, USA), following the GBS protocol (Elshire *et al.* 2011). The restriction enzyme ApeKI was chosen after optimisation. This library was sequenced in two lanes of an Illumina HiSeq to ensure sufficient coverage To detect sex-linkage in this study, we used only unrelated individuals (no known family structure), totalling 32 female and 27 male *S. latifolia*.

The bioinformatics for *Silene* genotyping was identical to that employed for *Nepenthes*, as outlined in the main text*,* i.e. *de novo* reference assembly following a modified dDocent pipeline (Puritz *et al.* 2014), read mapping, variant calling and quality filtering. We then applied the same methods for detection of sex-linkage as we did to *Nepenthes*.

*How do the sex-linked loci we discover in* S. latifolia *compare to other reports?*

We compared our methods and results for *S. latifolia* to contigs with known sex-linkage status from the literature, which were mainly discovered by pedigree analyses. These included 25 genes from a linkage map (Bergero *et al.* 2013), 16 sex-linked BACs (Blavet *et al.* 2015), and 46,178 transcripts (Muyle *et al.* 2018), of which 1,105 were sex-linked. All RAD-tags (contigs) were searched via BLAST against these literature contigs and hits accepted with at least 90% of the length of the RAD-tag aligned and e-value 1e-5, resulting in 43,748 matches (3.5% of all RAD-tags), which were then used to make comparisons. Following Muyle *et al.* (2016), we calculated three measures of statistical power:

- true positive rate (TPR, sensitivity) = true positives / (true positives + false negatives)
- true negative rate (TNR, specificity) = true negatives / (true negatives + false positives)
- false discovery rate (FDR) = true positives / (true positives + false positives)

In samples from wild populations, we found 2,376 SNPs associated with sex (XY-patterned SNPs) in 1,027 contigs (associated test in PLINK, Purcell *et al.* 2007), 289 (28%) of which were already known to be sex-linked. These reproduced 7% of previously described sex-linked contigs (true positive rate, Table S11) and correctly rejected sex-linkage in 99.7% of cases (true negative rate), while 33% of our XY-patterned contigs were previously not identified as such (false discovery rate). However, the FDR here does not specifically count errors but also includes contigs where previous studies failed to detect sex-linkage, for example because of insufficient variants, or because of missing data. The low sensitivity was expected, because the association test can detect only the most highly diverged X-Y SNPs (frequency 0.5 in males and 1.0 in females), whereas segregation analysis can detect sex-linkage for any SNP in the parental genotypes, independent of their population allele frequencies. Our many novel sex-linked contigs are likely not errors but were undetectable in previous studies, because we analysed GBS data (subset genomic DNA) while previous studies targeted mostly expressed genes.

We then searched the *Silene* data for sex-specific contigs with privacy rarefaction. The XY sex chromosome system was correctly diagnosed, as expected (see main text). Furthermore, there were several matches between our male-specific (Y-hemizygous) *S. latifolia* contigs (32/593 = 5%, stringency eleven) and previously identified sex-linked contigs. These suggest a sensitivity of 0.6%, a specificity of 99.9%, and an FDR 56% (Table S11). However, no matches were expected because the previously known sex-linked *S. latifolia* contigs should have X and Y copies, or only X copies (X-hemizygous), as they were mostly identified by pedigree analyses, which requires presence in both sexes.

This suggests two aspects: First, Y-hemizygosity may be underexplored in *S. latifolia* relative to other classes of sex-linkage. Our rarefaction method considers only Y-hemizygosity (or W-hemizygosity) and aims to fill this gap among existing methods for the inference of sex-linkage. For example, SEX-DETector (Muyle *et al.* 2016) considers only XY genes and X-hemizygosity.

Second, we speculate that at least some of the Y-hemizygous contigs we identified may be interspersed with regions retaining high X-Y similarity, instead of forming a continuous region without similarity on the X. Due the library preparation method, GBS may isolate short Y-hemizygous windows, while other sequencing methods that access also flanking regions of such windows (BACs, RNA-seq, Sanger-seq) may allow to co-assemble the X and Y-alleles into a single, longer contig.

*Why does the proportion of sex-linked contigs we discover in* S. latifolia *appear relatively low?*

As described in the main text, we found around 1.6% of *S. latifolia* contigs to be XY-patterned, and another 1.52% to be male-specific (Y-hemizygous loci). However, it is estimated that c. 14% of the physical size of the female *S. latifolia* genome is comprised by the X chromosome (Matsunaga *et al.* 1994; Bergero & Charlesworth 2011), which may suggest a larger fraction of the genome to be sex-linked.

One of the most comprehensive datasets so far described (Muyle *et al.* 2018) detected sex-linkage for only 2.4% of expressed *S. latifolia* genes (1,105 / 46,178), which may thus be a more realistic expectation for the fraction of the *Silene* genome that is effectively sex-linked. The fraction detected by Muyle *et al.* (2018) is still higher than our result for XY-patterned loci, but rather close (our male-specific loci can effectively not be compared to Muyle *et al.*'s (2018) results, because they are not detectable by their approach). The lower proportion of XY-patterned contigs in our study can be explained by two factors, which are (a) the collapse of repetitive regions, which are known to be enriched on the sex chromosomes, into single contigs during the assembly of RAD-tags (contigs) and their exclusion from the dataset due to excessive read coverage, and (b) that our relatively small sample size limits detectability to SNPs with very high allele frequency differences between the sexes (i.e. near perfect divergence between X and Y alleles: frequency 0.5 in males and 1.0 in females). Segregation analysis (Muyle *et al.* 2018) in contrast can detect sex-linkage for any SNP in the parental genotypes, independent of their population allele frequencies

**Text S4 Privacy rarefaction**

*Problem statement*

Methods to rapidly genotype individuals across the genome, such as RAPDs, AFLPs, and more recently RNA-seq and ddRAD-seq have often been used to identify sex-specific markers. All these approaches suffer from the common problem that marker absence in some individuals (e.g. due to polymorphism, low coverage or technical artefacts) must be distinguished from true marker absence in the entire sex. Erroneous inference of marker absence in one sex leads to false positive sex-specific markers for the other sex. This likely played a role in studies reporting both male- and female-specific markers in single populations (Bewick *et al.* 2013; Gamble & Zarkower 2014; Heikrujam *et al.* 2015; Brelsford *et al.* 2017).

Theoretical expectations for populations of fully dioecious diploids, deviating from panmixia only by the mating system, imply that they can not harbour both male- and female specific loci at the same time. Under these assumptions, at most one sex, or else none, carries sex-specific alleles or loci as derived from the principles of diploid inheritance (Mendel 1866): If sex is determined by a single locus, here not understood as a single gene but more broadly as a non-recombining genomic region that may contain any number of genes together with the sex-determining gene(s), it must follow dominant-recessive inheritance (where absence constitutes a recessive allele). Co-dominance can be excluded because it would produce hermaphrodites or steriles, violating the assumption of a fully dioecious population. Consequently, all loci that are not perfectly physically linked to the sex-determining locus are expected to be shared by both sexes, and all sex-specific loci and alleles must be located in a non-recombining genomic region (either a single, physically continuous region, or multiple co-segregating chromosomes) that includes a dominant sex-determining allele. Even in the unusual XYW system (Orzack *et al.* 1980) or XY-ZW transitional phases (Sander van Doorn & Kirkpatrick 2010), only one sex has a sex-specific, since dominant, sex chromosome. If sex is controlled by more than one locus, as in quantitative or polygenic sex-determination, however, by definition no single locus or allele controls sex, and hence all loci and alleles are expected to be shared by both sexes.

*Privacy rarefaction procedure*

We define sex-specific loci as contigs to which sequencing reads can be aligned from one of the sexes only. The number and identity of sex-specific contigs both carry uncertainties because they depend on the number and identity of male and female individuals compared. We test sex-specificity quantitatively, i.e. for deviation of the observed number of sex-specific contigs from zero, and qualitatively for each locus to retain only the best candidates for downstream analyses.

The privacy rarefaction procedure subsamples male and female individuals at a 1:1 sex ratio to allow unbiased comparisons among data sets with different total numbers of individuals and sex ratios. To capture the uncertainty over different combinations of individuals, n random sets of males and females are bootstrapped without replacement for subsample size 1 to the maximum possible subsample size, defined as the minimum of (N males, N females). We also refer to the subsample size, or number of individuals of each sex, as the 'stringency', because higher subsample sizes imply a greater confidence in the result. In the quantitative tests, sex-specific loci are counted for each combination. This generates two distributions, the observed (but boostrap-resampled) male- and female-specific loci counts. Then, these two distributions are separately compared to a null distribution derived from permutations of the sexes. The null distribution estimates how many loci would falsely appear to be sex-specific if the individuals were interchangeable.

The qualitative test assesses the confidence in sex-specificity for each locus. Again, n random sets of males and females are boostrapped without replacement from the available individuals, for each possible subsample size. Then, for each locus, we count the number of bootstrapped male–female comparisons in which it appeared as sex-specific. True sex-specific loci are expected to appear more frequently in such comparisons than false positives whose occurrence is random. The bootstrap support value for sex-specificity is the count divided by the number of bootstraps.

The following lines present the quantitative and qualitative procedures of privacy rarefaction as pseudocode:

––––––––––––––––––––––––––––––––––––––––––––––––––––––––––––––––

quantitative assessment of sex-specificity:

for n in 1... [minimum sample size of the two sexes]: # subsampling, or 'stringency'

repeat n_resampling times: # bootstraps

1. take random subsamples of size n from each males and females
2. get observed number of male-specific and female-specific loci
3. female specific count: count the loci that do map female reads but not male reads (cardinality of set difference F \ M )
4. male specific count: count the loci that do map male reads but not female reads (cardinality of set difference M \ F )
5. store the two counts
6. get permuted number of male-specific and female-specific loci
7. permute the sexes: male and female are mixed
8. count the loci that do map group A reads but not group B reads (cardinality of set difference A \ B )
9. count the loci that do map group B reads but not group A reads (cardinality of set difference B \ A )
10. store the two counts

Then: compare the observed and permuted (null) distribution. If the two distributions overlap, the number of apparent sex-specific loci is explicable by random sampling from a homogenous population, and thus not significant. A p-value indicates the proportion of permuted sex-specific counts that are equal to or larger than the mean of the observed sex-specific count distribution.

––––––––––––––––––––––––––––––––––––––––––––––––––––––––––––––––

qualitative assessment of sex-specificity:

for n in 1... [minimum sample size of the two sexes]: # subsampling, or 'stringency'

repeat n_resampling times: # bootstraps

1. take random subsamples of size n from each males and females
2. female specific loci: find the set of loci that do map female reads but not male reads (set difference F \ M)
3. male specific loci: find the set of loci that do map male reads but not female reads (set difference M \ F)
4. store these two sets of loci

Then: count bootstrap support value per locus

for each locus:

1. count the number of resampled F sets that contain the locus
2. count the number of resampled M sets that contain the locus

––––––––––––––––––––––––––––––––––––––––––––––––––––––––––––––––

––––––––––––––––––––––––––––––––––––––––––––––––––––––––––––––––

*Notes & Discussion*

We argue that erroneous inference of marker absence in one sex, and thus false positive identification of sex-specific markers in the other sex, results from insufficient consideration of uncertainty in presence–absence within and between sexes. This may include sex bias in both sample size, and genetic structure of the population screened. We eliminated this problem through replicated downsampling from a larger pool of observations (individuals) in the same way as rarefaction in community ecology eliminates sampling bias when comparing species richness (Gotelli & Colwell 2001). However, instead of the resampled counts in two groups (habitats), we record the identity and level of sharing (or privacy) between groups (sexes), which is not of interest in conventional rarefaction analysis. As a result, unlike conventional rarefaction analysis, privacy rarefaction curves decline towards a plateau with increasing sub-samplesizes, rather than increase.

An empirical statistical solution to differentiate between random and true sex-specificity of genomic sequences or variants has to our knowledge not been applied before (but see Kalinowski 2004; Schlüter & Harris 2006; Szpiech *et al.* 2008 for applications in genetic fingerprinting and diversity estimation). Previous approaches to detect sex-specific loci from wild populations sequenced several males and females and then scored which loci are absent from all males and present in all females, and vice-versa. The scoring is done naively, and only once with all individuals together, frequently with different sample sizes for males and females. The privacy rarefaction curves in Fig. 2 of the main text show false positives in at least one sex (these are apparent sex-specific loci identified in both sexes, typically at low stringency), especially when too few males and females are compared. With just two individuals of each sex, we typically obtained tens of thousands of candidate sex-specific contigs, most of which were false positives. With increasing numbers of males and females, false positives are progressively eliminated as the observed male and female sets of loci accumulate and approach completeness. Different combinations of many males and many females yield small but largely consistent sets of sex-specific candidates. In our experietruence, this required at least ten individuals of each sex, and our script reports this consistency as a bootstrap support value. The dropout of false positives produces the initial steep decay of the privacy rarefaction curves and continues until only one sex contains sex-specific loci, identifying the heterogametic sex. We expect that the sex-specific candidates that occur at and beyond this stringency have a near zero false discovery rate, because there are no more false positives for the homogametic sex. Tests on simulated data confirm the absence of false positives at high levels of stringency, but also imply a high false negative rate, arising from missing data (Text S5). True sex-specific loci will be classified as not sex-specific if they are absent in a subset of the investigated individuals of the heterogametic sex. The slower decay of privacy rarefaction curves at higher stringencies is due to the dropout of true positive candidates.

As the noisy individual presence-absence is smoothed by aggregation over many individuals, one may argue that sex-specificity should be scored only for the maximum possible number of males and females in a dataset. However, this strategy can miss true sex-specific loci entirely if the data are noisy and true sex-specific loci have inconsistent and low coverage, e.g. when large genomes with very small sex-specific regions are sequenced at low depth. But if combinations of fewer males and females are also evaluated, it is possible to find that one sex consistently yields more candidates than the other, which is sufficient to infer that true positives exist for that sex. Our script calculates a p-value for the difference between male- and female-specific candidate counts. Importantly, the privacy rarefaction algorithm per se can not affect the false negative rate because it is not based on statistical model assumptions - false negatives are given by stochasticity in read presence-absence, i.e. due to the sampling design, wetlab and in-silico procedures.

A recent review discussed six available methods for identifying sex-linked sequences (Muyle *et al.* 2017). Our method, the combination of population genomic data (individual data generated by any sequencing method, e.g. RNA-seq, reduced-representation libraries, whole-genome) with privacy rarefaction, fills a gap in these existing methods, most importantly because it does not depend on breeding or availability of an assembled reference genome. Some of these methods further require prior knowledge of the heterogametic sex and exhaustive genome sequencing of males and females. Our method is related to the Bayesian classification algorithm detsex (Gautier 2014) in some of its scope and the required input data. However, privacy rarefaction is model-free, was here used successfully with less than half of the individuals recommended for detsex (i.e. 10-20 per sex as opposed to >40; Muyle *et al.* 2017), and copes with very large and noisy datasets with high missingness. Privacy rarefaction curves thus offer a unique, simple and robust way to judge whether true sex-specific loci exist and which sex is heterogametic. The established methods for identification of sex-linked sequences are more suitable for projects that aim to further study previously identified sex chromosomes, while our approach is efficient in the first phase of investigation of new species, i.e. rapid *de novo*-discovery of unknown sex determination systems and cytogenetically homomorphic sex chromosomes with some of their basic properties. Privacy rarefaction, however, can also complement more advanced projects, because it identifies sex-specific loci (Y- resp. W-specific loci), a major class of sex-linkage that is neglected by family segregation analyses due to the lack of recombination.

A caveat of using population polymorphism is that it can be confounded by deviation from panmixia due to factors other than the mating system, namely family structure and geographic population structure in the sample. For example, in a species with XY sex-determination, full-sibling males and females do not contain identical X-chromosomes if their parents carried polymorphic X chromosomes; this may lead to the apparent paradox of finding both male- and female-specific markers. Ideally, individuals should be sampled from a single deme. Lastly, the resampling strategy should make this method robust to modest levels of erroneous sex assignment.

Privacy rarefaction is implemented as a multithreading python script that calls samtools (Li *et al.* 2009) to read mapping data from .bam alignments. The code and a tutorial are available at https://github.com/mscharmann/privacy-rarefaction.

**Text S5 Performance analysis of privacy rarefaction on simulated RAD-data**

*Simulations*

We assessed the power to detect sex-specific loci with RAD-data and privacy rarefaction using simulations. These replicated the missing data patterns in RAD-data, but did not consider population genetic properties such as polymorphism and genetic structure. The simulations thus cover only technical presence-absence artefacts. For simplicity, only ZW scenarios were simulated but the same applies to XY scenarios. We assumed that true biological presence-absence in the form of female-specific loci is overlain by random artefactual presence-absence in a RAD-like fashion. The total number of loci was kept at 10,000. To represent noise in RAD-data such as allele dropout and PCR-bias, each locus was assigned an intrinsic probability of being sequenced between 0.05 to 1, sampled from a truncated exponential decay distribution. A further stochastic element was the coverage of each sample, i.e. the number of sequenced loci.

Parameters explored in the simulations were as follows:

- sampling scheme (balanced: 20 males and 20 females, male-bias: 20 males and 10 females, female-bias: 10 males and 20 females)
- number of female-specific loci (1, 10, 100, 1000)
- coverage / number of loci sequenced per sample, sampled from a normal distribution (low coverage: mean 4,500 with standard deviation 900, high coverage: 9,000 with standard deviation 1,800)

For each parameter combination we ran 50 simulations, and we measured whether females were correctly inferred as the heterogametic sex, and whether sex-specific loci were identified as such. The privacy rarefaction method was contrasted with a naive scoring method, where female-specific loci are identified simply by their presence in at least one female sample and absence in all males.

*Results and Discussion*

On idealistic data (high coverage, balanced sampling of males and females), both rarefaction and naive scoring nearly always correctly inferred females as the heterogametic sex (Figure S5-1 panels A,C,E). However, on more realistic low-coverage data, naive scoring always failed (S5-1 panels B,D,F) because it identified both male- and female-specific loci, which is biologically implausible and counter to the simulated truth. Rarefaction showed a clear advantage with >90% success when at least one permil of loci were sex-specific (Figure S5-1 B). Furthermore, sex-biased sampling schemes confounded the naive scoring, whereas rarefaction generally succeeded when at least 1-10 permil of loci were sex-specific (Figure S5-1 C,D,E,F).

Regarding the identification of female-specific loci (simulated truth), naive scoring recovered close to 100% of the true female-specific loci (true positives), but in low-coverage data it reported in addition large numbers of false positives (Figure S5-2). Furthermore, naive scoring always identified false male-specific loci in low coverage data (Figure S5-3). True positives can not be distinguished from false positives by naive scoring, thus results of naive scoring are of limited value. Rarefaction in contrast almost never reported false positives, independent of the coverage (Figures S5-2, S5-3). In practice, false positives can be fully excluded by inspection of the output files and manual determination of the stringency threshold, which was kept very low in the simulations: two individuals of each sex. However, the near-zero false discovery rate of the rarefaction method came at the cost of low true positive rates, ranging around 20% for low-coverage data (Figure S5-2).

We also assessed how accurately privacy rarefaction estimated the proportion of sex-specific contigs in the dataset, as this can provide a measure of the relative size of sex-specific regions in a genome. This proportion was here measured as the number of female-specific contigs divided by the total number of contigs in the females, at stringency level 10. As expected, the accuracy of this prediciton was better for higher coverage, more female-specifc regions, and balanced sampling schemes (Text S5 Table S5-1). When the true proportion of female-specific contigs was at least 1 permil, the prediction error always remained <=76% (except for male-biased sampling at low coverage). This suggests that the relative size of sex-specific regions in the genome can be estimated to the correct order of magnitude in most cases. The loss of autosomal and sex-specific contigs with increasing stringency appears to be proportional, as can be expected given our simulations under a shared distribution of the probability to be sequenced.

In conclusion, privacy rarefaction on RAD-like data makes almost no errors in the detection of the heterogametic sex and identification of sex-specific loci, but it is likely to miss many more sex-specific loci. Clearly, the power to detect a sex-specific region depends on its relative size. On the base of our simulations, we estimate that privacy rarefaction with RAD-data provide a >90% chance to correctly identify some sex-specific loci when at least 0.1 permil - 1 permil of the sequenced contigs are sex-specific. For example, a typical RAD-type protocol may generate upwards of 100,000 contigs from a 1 Gb genome for 20 males and 20 females, and would almost certainly detect a sex-specific region if it is at least on the order of 100 kb to 1 Mb in physical size. A caveat of our method is that higher densities of markers would be required for dioecious species with even smaller sex-specific regions. Some species may also have only XY- or ZW-patterned regions (sex chromosomes that only differ in allele frequency but not locus presence-absence), and hence one would have to rely on SNP genotyping and association tests.


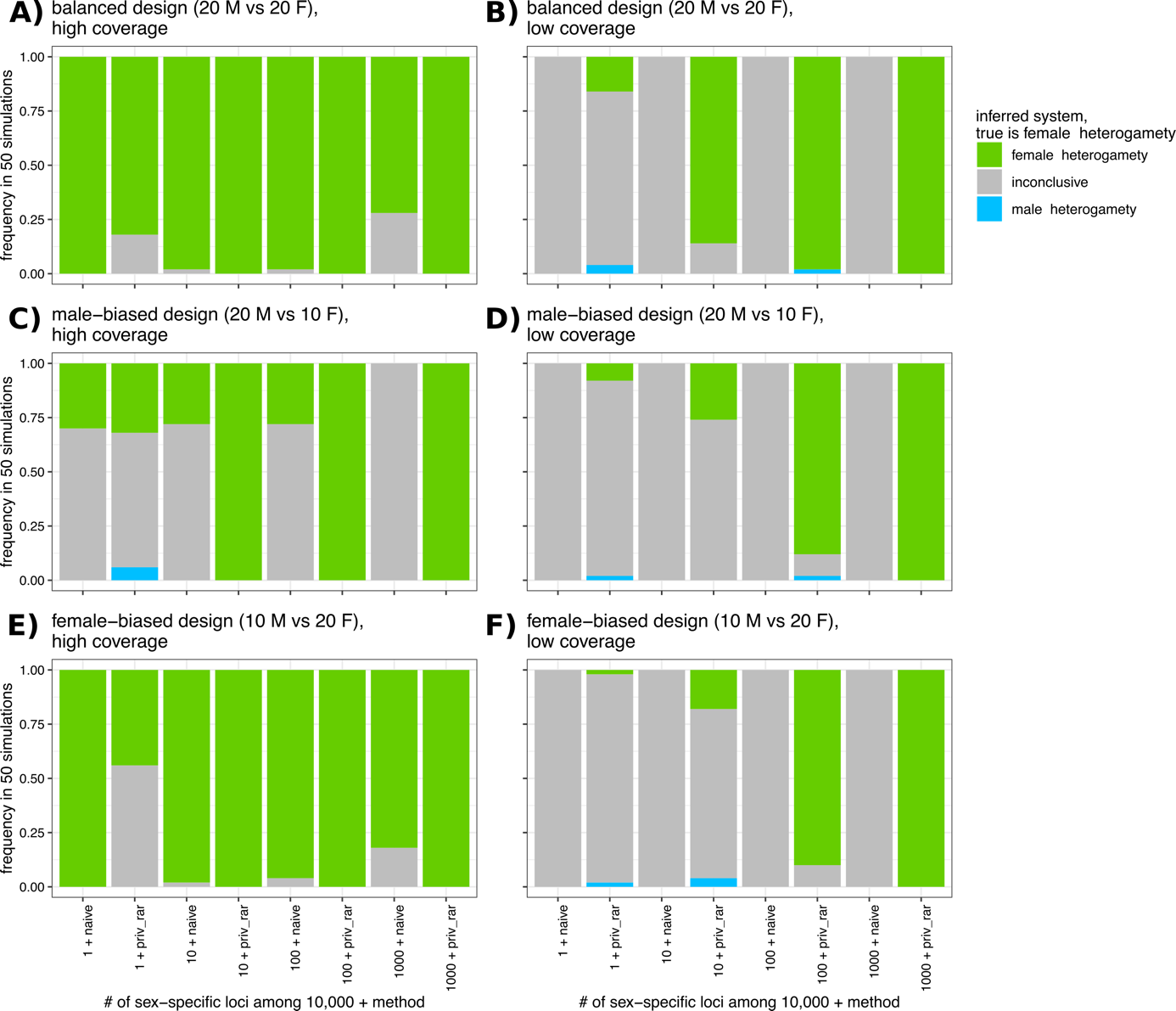


Figure S5-1. Power of privacy rarefaction and a naive scoring method for inference of female heterogamety (ZW sex chromosome system) in RAD-like data. Shown is the proportion of correct, inconclusive and wrong inferences among 50 independent simulated datasets with identical parameters (Y-axis), in dependence of the number of female-specific contigs (X-axis; either 1, 10, 100 or 1,000 among 10,000 total contigs), coverage (high / panels A,C,E = each sample has read coverage on avg. 90% of 10,000 contigs, low / panels B,D,F = each sample has sequenced on avg. 50% of 10,000 contigs), and the sampling scheme (A,B balanced; C,D male-biased; E,F female-biased).


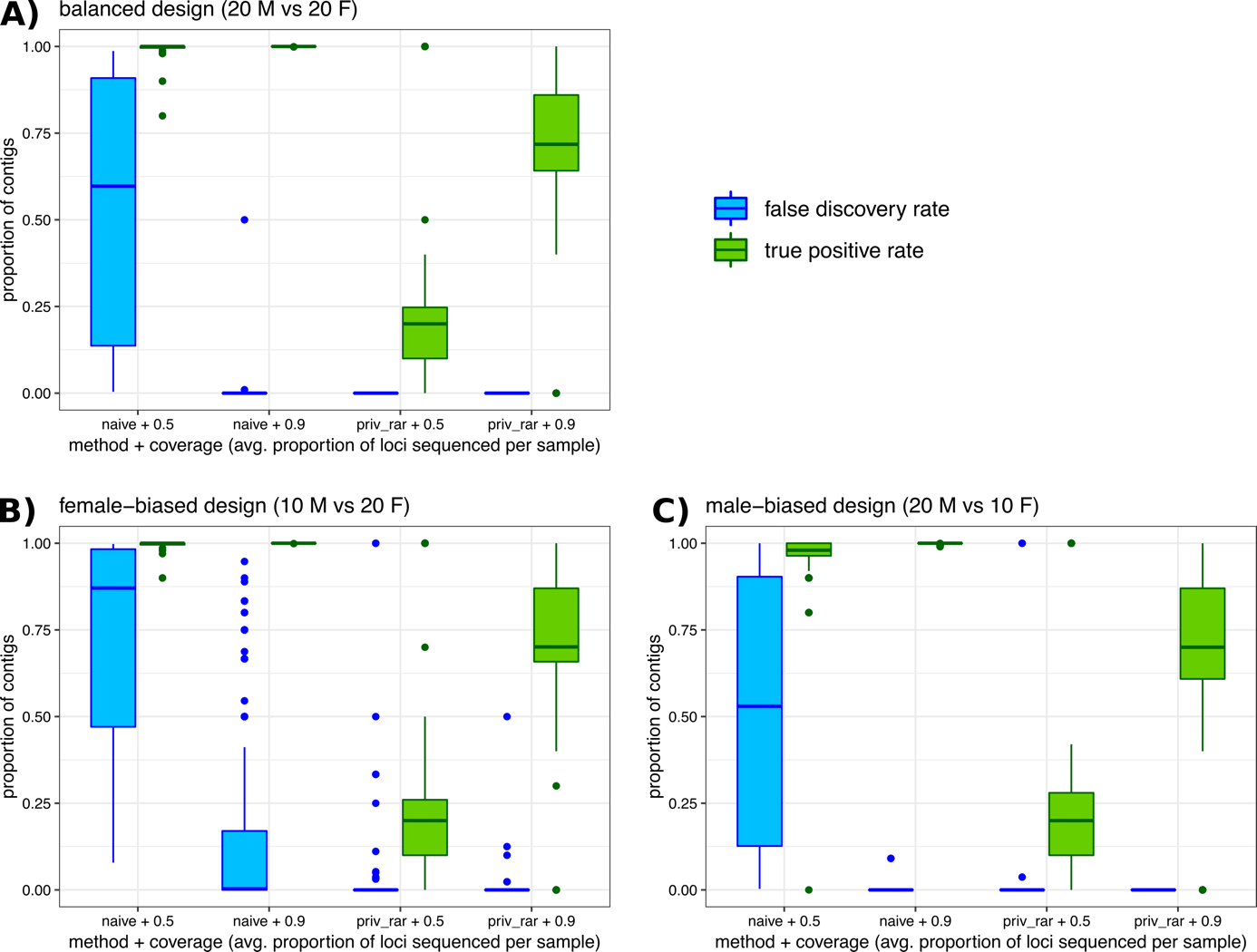


Figure S5-2: Power of privacy rarefaction and a naive scoring method for discovery of female-specific (W-hemizygous) contigs in RAD-like data. Shown are the false discovery rate (autosomal contigs among all contigs classified as female-specific), and true positive rate (proportion of true female-specific contigs recovered as such, sensitivity), for high and low coverage datasets (high = each sample has sequenced on avg. 90% of 10,000 contigs, low = each sample has sequenced on avg. 50% of 10,000 contigs). Each box contains results from 200 independent simulations, with either 1, 10, 100 or 1,000 female-specific contigs in 10,000 total contigs. A) balanced sampling scheme, B) female-biased sampling scheme, c) male-biased sampling scheme.


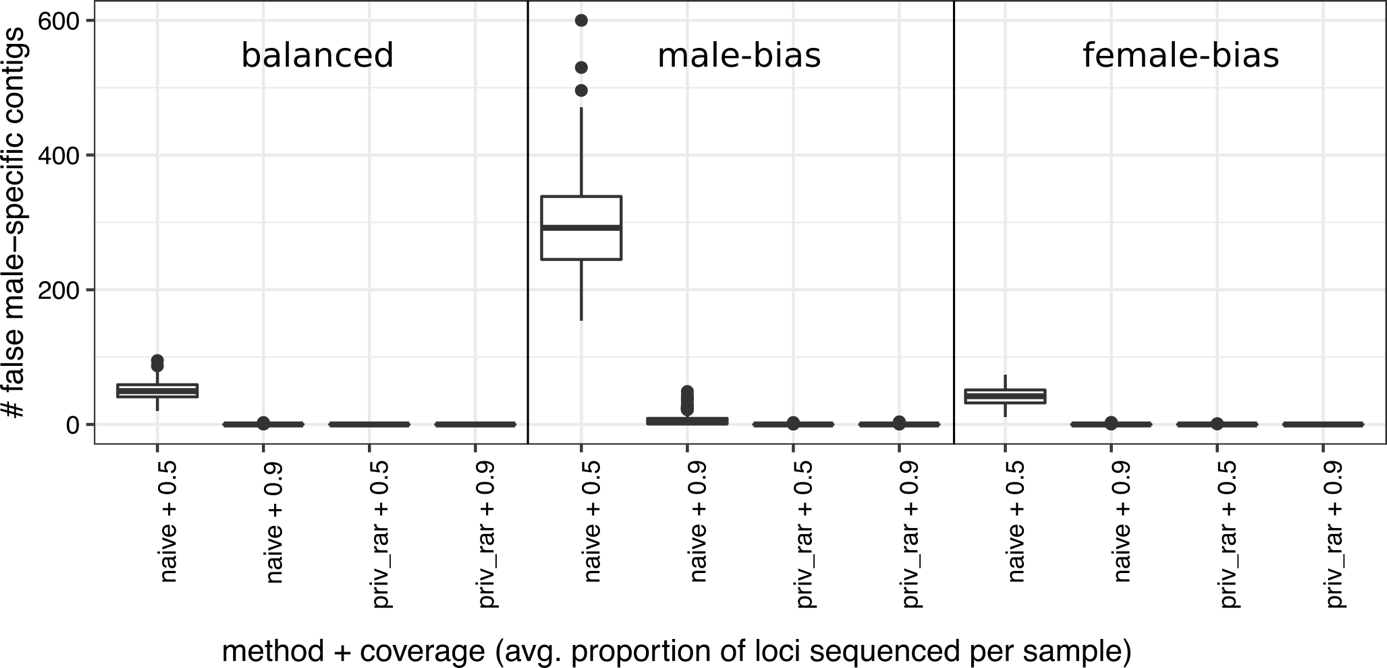


Figure S5-3. False male-specific contigs reported by privacy rarefaction and naive scoring for data simulated with only autosomal and female-specific contigs. Coverage and sampling design as above. 200 simulations per box.

Table S5-1. Accuracy of the prediction of the relative proportion of female-specific contigs in the dataset by privacy rarefaction. Shown is the mean absolute percentage error (MAPE), i.e. the average over the absolute differences between true and estimated values divided by the true values. Results are based on 50 simulations for each combination of sampling scheme, coverage, and true proportion of female-specific contigs, and contig counts at stringency level ten.

| **sampling scheme** | **coverage** | **true prop. female-specific** | **MAPE** |
| --- | --- | --- | --- |
| balanced | 0.5 | 0.1 | 7 |
|  |  | 0.01 | 26 |
|  |  | 0.001 | 76 |
|  |  | 0.0001 | 99 |
|  | 0.9 | 0.1 | 4 |
|  |  | 0.01 | 9 |
|  |  | 0.001 | 33 |
|  |  | 0.0001 | 100 |
| female-bias | 0.5 | 0.1 | 7 |
|  |  | 0.01 | 24 |
|  |  | 0.001 | 75 |
|  |  | 0.0001 | 141 |
|  | 0.9 | 0.1 | 3 |
|  |  | 0.01 | 9 |
|  |  | 0.001 | 30 |
|  |  | 0.0001 | 93 |
| male-bias | 0.5 | 0.1 | 14 |
|  |  | 0.01 | 52 |
|  |  | 0.001 | 141 |
|  |  | 0.0001 | 220 |
|  | 0.9 | 0.1 | 5 |
|  |  | 0.01 | 10 |
|  |  | 0.001 | 32 |
|  |  | 0.0001 | 141 |

**Text S6 Male inflorescence transcriptome of *N. khasiana***

Plants of *Nepenthes khasiana* (*in vitro* propagated material from Borneo Exotics (Pvt) Ltd., Sri Lanka) were grown in a greenhouse where they flowered regularly. For the transcriptome of a male inflorescence of *N. khasiana* (length c. 2 cm, many buds of c. 1-3 mm diameter each), we extracted RNA using the Total RNA Mini Kit (Plant) (Geneaid Biotech Ltd, New Taipei City, Taiwan) with the "PRB" lysis buffer, which yielded undegraded high quality RNA (RIN 7.1; Plant RNA Nano Assay, Agilent Bioanalyzer). A cDNA library was generated (NEBNext Ultra Directional RNA Library Prep Kit for Illumina, New England Biolabs, Ipswich MA, USA) and sequenced in one lane of the Illumina MiSeq for 150 bp paired-end reads (GDC ETHZ). A total of 18.7 million PE reads were obtained, and a reference transcriptome was *de novo* assembled by the Trinity pipeline (Grabherr *et al.* 2011). Best ORFs were extracted by the Transdecoder script.

**Text S7 Phylogenetic dating of *Nepenthes***

*Materials and method*

We dated the genus *Nepenthes* by combining new transcriptome data for *Nepenthes* with previously published transcriptome data of the Venus Flytrap (*Dionaea muscipula*, Bemm *et al.* 2016), a transcriptome-based phylogeny of Caryophyllales (Yang *et al.* 2015), and dates of Angiosperm diversification (Magallón *et al.* 2015). *Dionaea* represents Droseraceae, which is among the closest living sister lineages of *Nepenthes* (Brockington *et al.* 2009; Soltis *et al.* 2011).

In the first step, transcriptomes of 12 *Nepenthes* spp. (Scharmann *et al.* unpublished) were assembled de-novo using Trinity (Grabherr *et al.* 2011). The raw assembly for *Dionaea* (v1.03) was downloaded from http://tbro.carnivorom.com/. We extracted candidate ORFs resp. peptide sequences with TransDecoder.LongOrfs v3.0.0 and TransDecoder.Predict. To reduce the sequence collections even further in a meaningful way, we retained only peptides that were similar (e-value <= 1e-5) to any gene from all available Eudicot plant genome assemblies (NCBI Genbank, accessed 6 June 2016).

In the second step, we emended a taxon-subset of the peptide sequence matrix for the 1,122 genes of (Yang *et al.* 2015) with orthologs from the *Dionaea* and *Nepenthes* transcriptomes. A custom python script was used to decompose the matrix by gene and taxon using the also available gene model file. The peptide sequences of "*Nepenthes alata* (WQUF)" were used to identify orthologs in the new transcriptomes by reciprocal best hit (blastp) with an e-value cutoff of 0.01. Third, a matrix was re-assembled with 21 of the original taxa and 13 newly added taxa, by globally re-aligning all ortholog peptide sequences of each gene using MUSCLE (Edgar 2004), and concatenation of the alignments. A new gene model file was generated in the process to allow partitioned analysis of the matrix. The new alignment contained the same number of genes as before (1,122) but was slightly longer than the original (550,076 instead of 504,850 amino acid characters, including variable and fixed positions), contained 34 taxa, and 21.7% gap characters. The 13 taxa we added showed very high sequence occupancy, each containing >1,000 of the original 1,122 genes of Yang *et al.* (2015).

The maximum likelihood tree was reconstructed with the same method and partitioned by genes as before (RAxML -m PROTCATWAG -q ; Yang *et al.* 2015). SH-like support was calculated using RAxML -f J option.

We then dated the divergence times on a pruned version of this tree (see below) using the RelTime algorithm (Tamura *et al.* 2012) as implemented in MEGA-CC (Kumar *et al.* 2012). RelTime is a non-Bayesian method for dating of phylogenentic trees that produces estimates similar to those from e.g. BEAST and MCMCtree, but it is orders of magnitudes faster and thus copes with genomics-scale alignments (Mello *et al.* 2017). The pruned tree contained only Brassicaceae (*Arabidopsis thaliana*) as the outgroup, and hence the alignment given to RelTime was also reduced with the same method as above (32 taxa, 550,360 sites, 22% gaps). We specified the WAG substitution model with 5 gamma-distributed rate categories and invariant sites. For 13 nodes that were also present in the Angiosperm time-tree of Magallón *et al.* (2015), we supplied absolute time calibrations in the form of upper and lower limits on age (Table S7-1).

Preliminary runs of RAxML and RelTime revealed that inclusion of Fabaceae, Rosaceae and Brassicaceae resulted in the same topology as retrieved by Yang *et al.* (2015), but enforcing these "Rosids" as a monophyletic outgroup caused RelTime to fit negative branch lengths near the root. However, reducing the outgroup to just Brassicaeae, RAxML found a rather different topology compared to Yang *et al.* (2015), and to this tree RelTime fitted negative branch lengths among the major lineages of Caryophyllales. Thus, to avoid biologically not interpretable negative branch lengths, we obtained a topology using the three outgroup taxa, but pruned this tree and the alignment to retain only Brassicaceae during RelTime dating.

*Results and discussion*

We retrieved largely the same topology as Yang *et al.* (2015) for our subset of Caryophyllales taxa, with full SH-like LRT support for all nodes (tree not shown). The only exception, a grouping of Sarcobataceae as sister to Nyctaginaceae instead of Phytoloccaceae, occured in a lineage distant to *Nepenthes*. *Nepenthes* was monophyletic and grouped as sister to Droseraceae (*Dionaea muscipula*). This carnivorous lineage was sister to Frankeniaceae-Plumbaginaceae-Polygonaceae as reported before (Yang *et al.* 2015). The stem age of *Nepenthes* was estimated at 71.1 (CI 44.2 - 98.0) Mya, when it split from its presumed sister Droseraceae. The crown of *Nepenthes* is marked by the most basal species *N. pervillei*, and estimated here at 17.7 (CI 11.0 - 24.3) Mya.

However, we interpret these time estimates with great caution. First, the identity of the closest living relative of *Nepenthes* has not yet reached a consensus. Candidates are the lineage of sticky-leaf carnivores *Drosophyllum* and *Triphyophyllum* and several tropical lianas that appear to have lost carnivory secondarily (Heubl *et al.* 2006; Renner & Specht 2011), and the Droseraceae (Brockington *et al.* 2009; Soltis *et al.* 2011), or *Nepenthes* may even be basal to both of these (Brockington *et al.* 2015). We focussed on the Droseraceae because this was the only family for which transcriptome data was available.

Second, the divergence times that we took from the literature (secondary calibrations) may change in the future, as these were based on few genetic loci, fossils may be re-interpreted, and estimation methods change.

All previous attempts of molecular dating in *Nepenthes* (Meimberg 2002; Merckx *et al.* 2015) involved a presumed *Nepenthes* pollen fossil from the European Eocene (c. 50 million years ago; Krutzsch 1985). However, the attribution of this fossil to an ancestor of recent *Nepenthes* is not justified ­­– it is larger than recent *Nepenthes* pollen but instead fits in the range of Droseraceae (Cheek & Jebb 2001). Thus, Krutzsch's pollen fossils are at best indicative of the European Eocene presence of some lineage with Droseraceae-Nepenthaceae affinity but do not imply an age for modern *Nepenthes*.

Table S7-1. Absolute time calibrations as constraining upper and lower boundaries for the RelTime analysis, taken from Magallón *et al.* (2015). These are 95% confidence limits for the age (in million years) of the most recent common ancestor (MRCA) of 13 pairs of plant families studied by both Yang *et al.* (2015) and Magallón *et al.* (2015).

| **MRCA of** | **min time** | **max time** |
| --- | --- | --- |
| Cactaceae, Portulacaceae | 15.08 | 48.15 |
| Cactaceae, Talinaceae | 18.75 | 53.24 |
| Caryophyllaceae, Amaranthaceae | 50.34 | 88.56 |
| Cactaceae, Molluginaceae | 54.22 | 84.99 |
| Phytolaccaceae, Sarcobataceae | 63.24 | 73.41 |
| Plumbaginaceae, Polygonaceae | 65.63 | 78.21 |
| Aizoaceae, Nyctaginaceae | 72.5 | 77.87 |
| Phrymaceae, Solanaceae | 77 | 103 |
| Frankeniaceae, Plumbaginaceae | 83 | 101 |
| Caryophyllaceae, Physenaceae | 90 | 101 |
| Caryophyllaceae, Simmondsiaceae | 95.44 | 105.53 |
| Caryophyllaceae, Solanaceae | 114 | 123 |
| Brassicaceae, Nyctaginaceae | 120.87 | 126.49 |

**References**

Bemm, F., Becker, D., Larisch, C., Kreuzer, I., Escalante-Perez, M., Schulze, W.X., *et al.* (2016). Venus flytrap carnivorous lifestyle builds on herbivore defense strategies. *Genome Res.*, 26, 812–825.

Bergero, R. & Charlesworth, D. (2011). Preservation of the Y Transcriptome in a 10-Million-Year-Old Plant Sex Chromosome System. *Current Biology*, 21, 1470–1474.

Bergero, R., Qiu, S., Forrest, A., Borthwick, H. & Charlesworth, D. (2013). Expansion of the Pseudo-autosomal Region and Ongoing Recombination Suppression in the Silene latifolia Sex Chromosomes. *Genetics*, 194, 673–686.

Bewick, A.J., Chain, F.J.J., Zimmerman, L.B., Sesay, A., Gilchrist, M.J., Owens, N.D.L., *et al.* (2013). A large pseudoautosomal region on the sex chromosomes of the frog *Silurana tropicalis*. *Genome Biol Evol*, 5, 1087–1098.

Blavet, N., Blavet, H., Muyle, A., Käfer, J., Cegan, R., Deschamps, C., *et al.* (2015). Identifying new sex-linked genes through BAC sequencing in the dioecious plant Silene latifolia. *BMC Genomics*, 16.

Brelsford, A., Lavanchy, G., Sermier, R., Rausch, A. & Perrin, N. (2017). Identifying homomorphic sex chromosomes from wild-caught adults with limited genomic resources. *Mol Ecol Resour*, 17, 752–759.

Brockington, S.F., Alexandre, R., Ramdial, J., Moore, M.J., Crawley, S., Dhingra, A., *et al.* (2009). Phylogeny of the Caryophyllales sensu lato: Revisiting hypotheses on pollination biology and perianth differentiation in the core Caryophyllales. *Int J Plant Sci*, 170, 627–643.

Brockington, S.F., Yang, Y., Gandia-Herrero, F., Covshoff, S., Hibberd, J.M., Sage, R.F., *et al.* (2015). Lineage-specific gene radiations underlie the evolution of novel betalain pigmentation in Caryophyllales. *New Phytol*, 207, 1170–1180.

Catchen, J., Hohenlohe, P.A., Bassham, S., Amores, A. & Cresko, W.A. (2013). Stacks: An analysis tool set for population genomics. *Mol Ecol*, 22, 3124–3140.

Cheek, M.R. & Jebb, M.H.P. (2001). Nepenthaceae. *Flora Malesiana*, 15, 1–157.

Cho, Y., Qiu, Y.-L., Kuhlman, P. & Palmer, J.D. (1998). Explosive invasion of plant mitochondria by a group I intron. *Proc Natl Acad Sci U S A*, 95, 14244–14249.

Edgar, R.C. (2004). MUSCLE: Multiple sequence alignment with high accuracy and high throughput. *Nucl Acids Res*, 32, 1792–1797.

Elshire, R.J., Glaubitz, J.C., Sun, Q., Poland, J.A., Kawamoto, K., Buckler, E.S., *et al.* (2011). A robust, simple Genotyping-by-Sequencing (GBS) approach for high diversity species. *PLOS ONE*, 6, e19379.

Gamble, T. & Zarkower, D. (2014). Identification of sex-specific molecular markers using restriction site-associated DNA sequencing. *Mol Ecol Resour*, 14, 902–913.

Gautier, M. (2014). Using genotyping data to assign markers to their chromosome type and to infer the sex of individuals: a Bayesian model-based classifier. *Mol Ecol Resour*, 14, 1141–1159.

Gotelli, N.J. & Colwell, R.K. (2001). Quantifying biodiversity: Procedures and pitfalls in the measurement and comparison of species richness. *Ecol Lett*, 4, 379–391.

Grabherr, M.G., Haas, B.J., Yassour, M., Levin, J.Z., Thompson, D.A., Amit, I., *et al.* (2011). Full-length transcriptome assembly from RNA-Seq data without a reference genome. *Nat Biotech*, 29, 644–652.

Heikrujam, M., Sharma, K., Prasad, M. & Agrawal, V. (2015). Review on different mechanisms of sex determination and sex-linked molecular markers in dioecious crops: a current update. *Euphytica*, 201, 161–194.

Heubl, G., Bringmann, G. & Meimberg, H. (2006). Molecular phylogeny and character evolution of carnivorous plant families in Caryophyllales - revisited. *Plant Biol*, 8, 821–830.

Hobza, R. & Widmer, A. (2008). Efficient molecular sexing in dioecious *Silene latifolia* and *S. dioica* and paternity analysis in F1 hybrids. *Mol Ecol Resour*, 8, 1274–1276.

Kalinowski, S.T. (2004). Counting alleles with rarefaction: private alleles and hierarchical sampling designs. *Conserv Genet*, 5, 539–543.

Krutzsch, W. (1985). Über *Nepenthes*-Pollen (alias “*Droseridites*” p.p.) im europäischen Tertiär. *Gleditschia*, 13, 89–93.

Kumar, S., Stecher, G., Peterson, D. & Tamura, K. (2012). MEGA-CC: Computing core of molecular evolutionary genetics analysis program for automated and iterative data analysis. *Bioinformatics*, 28, 2685–2686.

Li, H., Handsaker, B., Wysoker, A., Fennell, T., Ruan, J., Homer, N., *et al.* (2009). The Sequence Alignment/Map format and SAMtools. *Bioinformatics*, 25, 2078–2079.

Magallón, S., Gómez-Acevedo, S., Sánchez-Reyes, L.L. & Hernández-Hernández, T. (2015). A metacalibrated time-tree documents the early rise of flowering plant phylogenetic diversity. *New Phytol*, 207, 437–453.

Matsunaga, S., Hizume, M., Kawano, S. & Kuroiwa, T. (1994). Cytological analyses in *Melandrium album*: Genome size, chromosome size and fluorescence *in situ* hybridization. *Cytologia*, 59, 135–141.

Meimberg, H. (2002). *Molekular-Systematische Untersuchungen an den Familien Nepenthaceae und Ancistrocladaceae sowie verwandter Taxa aus der Unterklasse Caryophyllidae s. l. PhD Thesis*. Ludwig-Maximilians-Universität München, München, Germany.

Mello, B., Tao, Q., Tamura, K. & Kumar, S. (2017). Fast and accurate estimates of divergence times from big data. *Mol Biol Evol*, 34, 45–50.

Mendel, J.G. (1866). Versuche über Pflanzenhybriden. *Verhandlungen des naturforschenden Vereines in Brünn, Bd. IV für das Jahr 1865, Abhandlungen*, 3–47.

Merckx, V.S.F.T., Hendriks, K.P., Beentjes, K.K., Mennes, C.B., Becking, L.E., Peijnenburg, K.T.C.A., *et al.* (2015). Evolution of endemism on a young tropical mountain. *Nature*, 524, 347–350.

Muyle, A., Käfer, J., Zemp, N., Mousset, S., Picard, F. & Marais, G.A. (2016). SEX-DETector: A Probabilistic Approach to Study Sex Chromosomes in Non-Model Organisms. *Genome Biol Evol*, 8, 2530–2543.

Muyle, A., Shearn, R. & Marais, G.A. (2017). The evolution of sex chromosomes and dosage compensation in plants. *Genome Biol Evol*, 9, 627–645.

Muyle, A., Zemp, N., Fruchard, C., Cegan, R., Vrana, J., Deschamps, C., *et al.* (2018). Genomic imprinting mediates dosage compensation in a young plant XY system. *Nature Plants*, 4, 677.

Orzack, S.H., Sohn, J.J., Kallman, K.D., Levin, S.A. & Johnston, R. (1980). Maintenance of the three sex chromosome polymorphism in the Platyfish, *Xiphophorus maculatus*. *Evolution*, 34, 663–672.

Purcell, S., Neale, B., Todd-Brown, K., Thomas, L., Ferreira, M.A.R., Bender, D., *et al.* (2007). PLINK: A tool set for whole-genome association and population-based linkage analyses. *Am J Hum Genet*, 81, 559–575.

Puritz, J.B., Hollenbeck, C.M. & Gold, J.R. (2014). dDocent: A RADseq, variant-calling pipeline designed for population genomics of non-model organisms. *PeerJ*, 2, e431.

Renner, T. & Specht, C.D. (2011). A sticky situation: Assessing adaptations for plant carnivory in the Caryophyllales by means of stochastic character mapping. *Int J Plant Sci*, 172, 889–901.

Sander van Doorn, G. & Kirkpatrick, M. (2010). Transitions between male and female heterogamety caused by sex-antagonistic selection. *Genetics*, 186, 629–645.

Schlüter, P.M. & Harris, S.A. (2006). Analysis of multilocus fingerprinting data sets containing missing data. *Mol Ecol Notes*, 6, 569–572.

Soltis, D.E., Smith, S.A., Cellinese, N., Wurdack, K.J., Tank, D.C., Brockington, S.F., *et al.* (2011). Angiosperm phylogeny: 17 genes, 640 taxa. *Am J Bot*, 98, 704–730.

Szpiech, Z.A., Jakobsson, M. & Rosenberg, N.A. (2008). ADZE: A rarefaction approach for counting alleles private to combinations of populations. *Bioinformatics*, 24, 2498–2504.

Tamura, K., Battistuzzi, F.U., Billing-Ross, P., Murillo, O., Filipski, A. & Kumar, S. (2012). Estimating divergence times in large molecular phylogenies. *Proc Natl Acad Sci U S A*, 109, 19333–19338.

Yang, Y., Moore, M.J., Brockington, S.F., Soltis, D.E., Wong, G.K.-S., Carpenter, E.J., *et al.* (2015). Dissecting molecular evolution in the highly diverse plant clade Caryophyllales using transcriptome sequencing. *Mol Biol Evol*, 32, 2001–2014.
